# The role of inferior frontal junction in controlling the spatially global effect of feature-based attention in human visual areas

**DOI:** 10.1371/journal.pbio.2005399

**Published:** 2018-06-25

**Authors:** Xilin Zhang, Nicole Mlynaryk, Sara Ahmed, Shruti Japee, Leslie G. Ungerleider

**Affiliations:** 1 School of Psychology, South China Normal University, Guangzhou, Guangdong, China; 2 Guangdong Provincial Key Laboratory of Mental Health and Cognitive Science, South China Normal University, Guangzhou, Guangdong, China; 3 Laboratory of Brain and Cognition, National Institute of Mental Health, National Institutes of Health, Bethesda, Maryland, United States of America; McGill University, Canada

## Abstract

Feature-based attention has a spatially global effect, i.e., responses to stimuli that share features with an attended stimulus are enhanced not only at the attended location but throughout the visual field. However, how feature-based attention modulates cortical neural responses at unattended locations remains unclear. Here we used functional magnetic resonance imaging (fMRI) to examine this issue as human participants performed motion- (Experiment 1) and color- (Experiment 2) based attention tasks. Results indicated that, in both experiments, the respective visual processing areas (middle temporal area [MT+] for motion and V4 for color) as well as early visual, parietal, and prefrontal areas all showed the classic feature-based attention effect, with neural responses to the unattended stimulus significantly elevated when it shared the same feature with the attended stimulus. Effective connectivity analysis using dynamic causal modeling (DCM) showed that this spatially global effect in the respective visual processing areas (MT+ for motion and V4 for color), intraparietal sulcus (IPS), frontal eye field (FEF), medial frontal gyrus (mFG), and primary visual cortex (V1) was derived by feedback from the inferior frontal junction (IFJ). Complementary effective connectivity analysis using Granger causality modeling (GCM) confirmed that, in both experiments, the node with the highest outflow and netflow degree was IFJ, which was thus considered to be the source of the network. These results indicate a source for the spatially global effect of feature-based attention in the human prefrontal cortex.

## Author summary

Attentional selection is the mechanism by which relevant sensory information is processed preferentially. Feature-based attention plays a key role in identifying an attentional target in a complex scene, because we often know the features of the target but not its exact location. The ability to quickly select the target is mainly attributed to enhancement of responses to stimuli that share features with an attended stimulus, not only at the attended location but throughout the whole visual field. However, little is known regarding how feature-based attention modulates brain responses at unattended locations. Here we used fMRI and advanced connectivity analyses to examine human subjects as they performed either motion- or color-based attention tasks. Our results indicated that the visual processing areas for motion and color showed the feature-based attention effect. Effective connectivity analysis showed that this feature-based attention effect was derived by feedback from the inferior frontal junction, an area of the posterior lateral prefrontal cortex involved in many different cognitive processes, including spatial attention and working memory. Further modeling confirmed that the inferior frontal junction showed connectivity features supporting its role as the source of the network. Our results support the hypothesis that the inferior frontal junction plays a key role in the spatially global effect of feature-based attention.

## Introduction

Attentional selection is the mechanism by which a subset of incoming information is processed preferentially. Numerous studies have demonstrated that attentional selection can be based on a spatial location [1–7]. Alternatively, attention can also select specific features independent of their spatial locations [8]. Several studies have demonstrated that attending to different feature dimensions, such as motion and color, enhances the response of their specialized cortical modules, i.e., human middle temporal area (MT+) and V4, respectively [9–16]. In addition, other studies have shown that attention can also select specific features within a particular dimension, such as an orientation [17–19], a color [20,21], and a direction of motion [22–25]. Feature-based attention plays a key role in identifying and highlighting a target during visual search because we often know a target-defining feature but not its exact location.

The compelling evidence for the location-independent property of feature-based attention has come from its spatially global effect, as proposed by the “feature-similarity gain model” [23,26], whereby feature-based attention can modulate the gain of cortical neurons tuned to the attended feature not only at the attended location but throughout the visual field. Remarkably, this spatially global effect has been demonstrated in numerous psychophysical [17,20,27,28], neurophysiological [22–24,29], electroencephalographic (EEG) [21,30], magnetoencephalogram (MEG) [19,31], and functional magnetic resonance imaging (fMRI) [18,25,32] studies in both striate (V1) and extrastriate visual areas. Both V1 and all of the areas within extrastriate visual cortex (V2–V4 and MT) only respond to stimuli presented in the contralateral visual hemifield [33,34]. However, during feature-based attention, all of these retinotopically organized areas can be modulated when participants attend to a feature presented anywhere in the visual field. How does feature-based attention modulate neural responses in these brain areas at unattended spatial locations (i.e., the spatially global effect)?

It has been well established that, during spatially directed attention, the enhanced responses in striate and extrastriate visual areas result from top-down feedback from frontoparietal cortical areas [4,5,35–37], and previous neurophysiological [16,38–40] and brain imaging [13,41] studies have suggested that the frontoparietal network is also involved in the top-down control of feature-based attention in the attended location [5,8,42,43]. Moreover, previous neurophysiological studies have shown that some neurons in frontoparietal areas have very large receptive fields [38]. Although the receptive fields of these neurons are not centered in the ipsilateral hemifield, many do extend into the ipsilateral hemifield, especially with longer stimulus presentation times [44,45]. This may provide the underlying neural basis for the spatially global effect of feature-based attention. Thus, we hypothesized that the spatially global effect of feature-based attention in striate and extrastriate visual areas might result from top-down feedback from frontoparietal cortical areas.

To test this hypothesis, we performed an fMRI experiment and used effective connectivity analysis to examine which area was involved in the spatially global effect of feature-based attention in retinotopically organized visual areas as human participants performed motion- (Experiment 1, a speed discrimination task) and color- (Experiment 2, a luminance discrimination task) based attention tasks. Results indicated that, in both experiments, the respective visual processing areas (MT+ for motion and V4 for color) as well as early visual, parietal, and prefrontal areas all showed the classic feature-based attention effect, with neural responses to the unattended stimulus significantly elevated when it shared the same feature with the attended stimulus. Furthermore, effective connectivity analysis using dynamic causal modeling (DCM) showed that the spatially global effect in the respective visual processing (MT+ for motion and V4 for color), intraparietal sulcus (IPS), frontal eye field (FEF), medial frontal gyrus (mFG), and V1 was derived by feedback from the inferior frontal junction (IFJ). Complementary effective connectivity analysis using Granger causality modeling (GCM) confirmed that, in both experiments, IFJ showed the highest outflow and netflow degree in the network and thus was considered to be the source of the network. Together, our findings indicate a source for the spatially global effect of feature-based attention in the human prefrontal cortex.

## Results

### Behavioral performance

**Fig 1:**
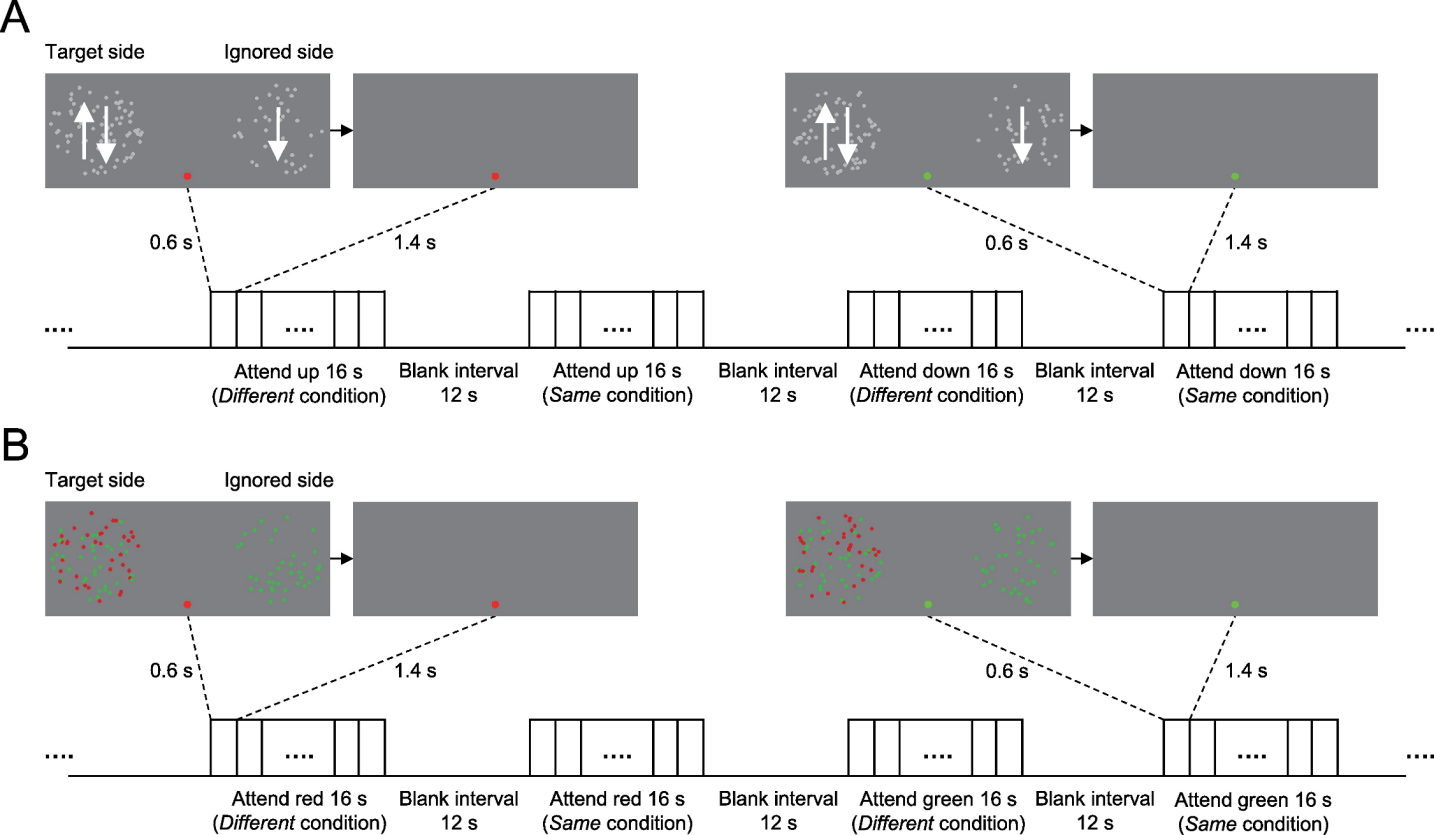
Sample stimuli and protocol of Experiments 1 and 2. The stimulus display in both Experiments 1 and 2 was composed of two circular regions in the upper visual field (centered 8.5° to the left and right of the central fixation spot). One of these regions was attended (the target side) and the other was unattended (the ignored side). (A) In Experiment 1, participants attended one direction of motion within a display of overlapping upward- and downward-moving dots (the attended stimulus) on the target side, and ignored a single field of dots moving either upward or downward (the unattended stimulus) on the ignored side. (B) In Experiment 2, the attended stimulus was a field of red or green stationary dots within a display of overlapping red and green stationary dots on the target side, and the unattended stimulus was a single field of red or green dots on the ignored side. On each trial, the stimulus was presented for 0.6 s, followed by a fixed 1.4-s fixation interval, and participants did a 0.2-s speed or luminance discrimination at threshold in Experiments 1 and 2, respectively.

Using a block design, Experiments 1 and 2 aimed to measure the feature-based attention effect as human participants performed motion- (Experiment 1) and color- (Experiment 2) based attention tasks, respectively. Both Experiments 1 and 2 consisted of six functional runs. Each run consisted of eight stimulus blocks of 16 s, interleaved with eight blank intervals of 12 s. There were four different stimulus blocks: 2 (attended feature: Upward/Downward in Experiment 1; Red/Green in Experiment 2) × 2 (feature match: Same/Different). In the Same condition, the feature on the ignored side matched the attended feature on the target side (half the blocks); a Different condition was defined as a mismatch (half the blocks) (Fig 1). Each stimulus block was randomly repeated two times in each run, and consisted of eight trials. On each trial, the stimulus was presented for 0.6 s, followed by a 1.4-s fixation interval. For attentional control, participants needed to press one of two buttons to indicate a 0.2-s speed and luminance change (increase or decrease) of the attended stimulus in Experiments 1 and 2, respectively. The speed and luminance changes were determined by QUEST [46] before scanning to ensure that participants performed equally well for the Same and Different conditions. The change detection thresholds, response accuracies, and reaction times for the Same and Different conditions in Experiments 1 and 2 are shown in S1 Fig. Paired *t* tests revealed that there was no significant difference (all *p* > 0.05) in all these measurements between the Same and Different conditions in either Experiments 1 or 2.

### Region of interest analyses

**Fig 2:**
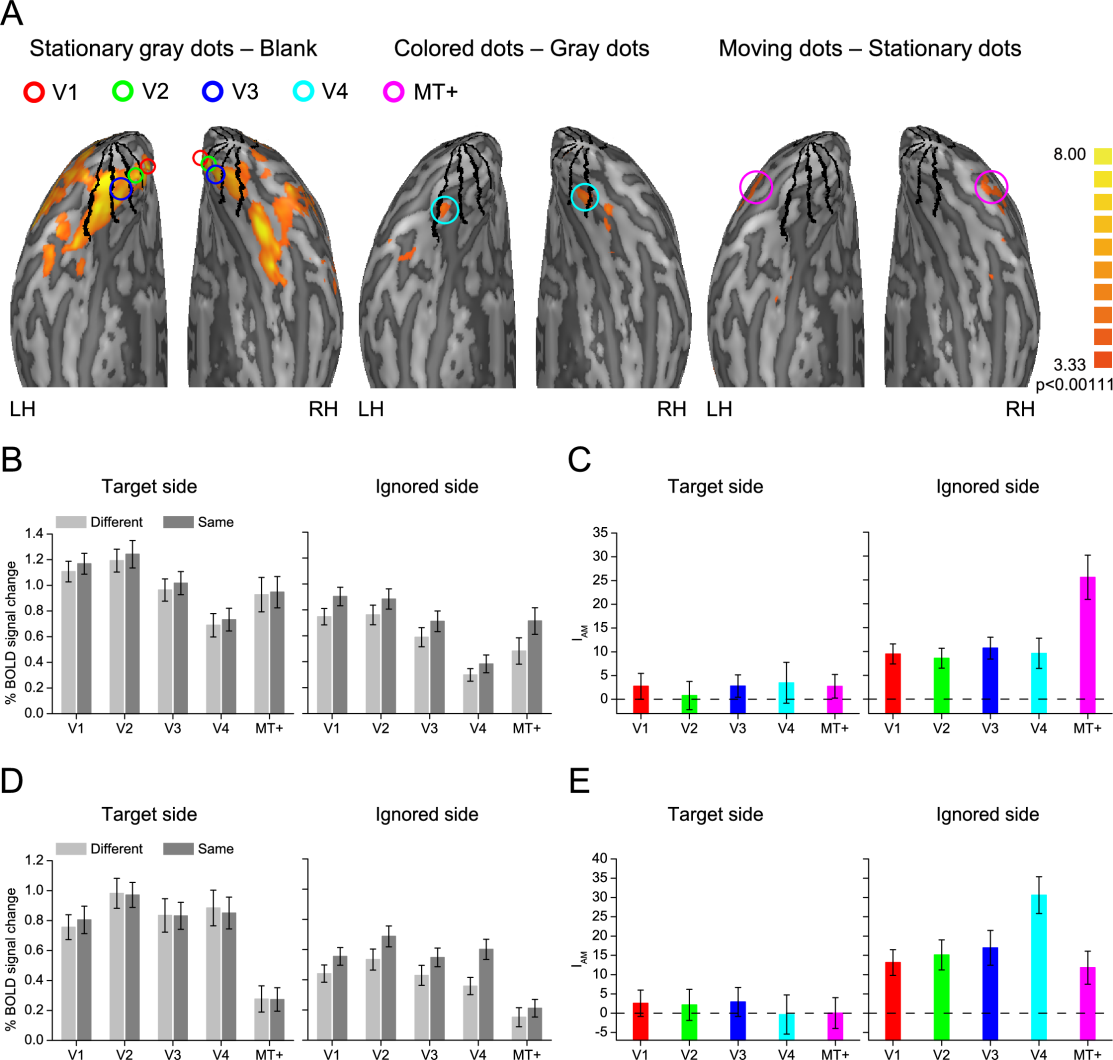
ROI analysis of Experiments 1 and 2. (A) ROIs on an inflated cortical surface of a representative participant. The ROIs in V1–V3 were defined as regions that responded more strongly to the stationary gray dots than to the blank screen. The ROI in V4 was defined as regions that responded more strongly to the stationary colored dots than to the stationary gray dots. The ROI in MT+ was defined as regions that responded more strongly to the moving dots than to the stationary dots. The boundaries among V1–V4, defined by retinotopic mapping, are indicated by the black lines. (B) BOLD amplitude of ROI in V1–V4 and MT+ evoked by stimuli on the target side (left) and ignored side (right) during the Same and Different conditions in Experiment 1. (C) I_A_ for the target side (left) and ignored side (right) of V1–V4 and MT+ in Experiment 1. (D) BOLD amplitude of ROI in V1–V4 and MT+ evoked by stimuli on the target side (left) and ignored side (right) during the Same and Different conditions in Experiment 2. (E) I_A_ for the target side (left) and ignored side (right) of V1–V4 and MT+ in Experiment 2. Error bars denote 1 SEM calculated across 19 participants. Data are available from the Open Science Framework (https://osf.io/8gqk6/). BOLD, blood oxygenation level–dependent; I_A_, attentional modulation index; LH, left hemisphere; MT+, middle temporal area; RH, right hemisphere; ROI, region of interest; V1, primary visual cortex.

Regions of interest (ROIs) in V1–V4 and MT+ were defined as the cortical regions responding significantly to the stimulus corresponding to the target and ignored sides of the display (Fig 2A). Blood oxygenation level–dependent (BOLD) signals were extracted from these ROIs and then averaged according to the feature match (the Same and Different conditions). For each stimulus block, the 2 s preceding the block served as a baseline, and the mean BOLD signal from 5 s to 16 s after stimulus onset was used as a measure of the response amplitude. For each participant, and for each side of the display (the target and ignored sides) and each ROI, we computed an attentional modulation index (I_A_) to quantify how much the measured response increased during the Same condition relative to the overall response to the stimuli in the ROI. The index was calculated as follows: I_A_ = (A _*Same*_ − A _*Different*_) / (A _*Same*_ + A _*Different*_)*100%, where A _*Same*_ and A _*Different*_ are the mean response amplitudes (A) in the Same and Different conditions, respectively. We hypothesized that if a cortical area shows a feature-based attention effect, the area should show a higher response in the Same condition than that in the Different condition. The I_A_ of this area then should be significantly higher than zero. However, if the cortical area does not show the feature-based attention effect, the I_A_ should not be significantly different than zero. Fig 2 shows the mean BOLD amplitudes in the Same and Different conditions and the corresponding I_A_ of the target and ignored sides. For the target side, V1–V4 and MT+ did not show a significantly higher response in the Same condition than that in the Different condition (Fig 2B and 2D, left), and none of these areas showed an I_A_ significantly different than zero in either Experiment 1 (V1: t_18_ = 1.014, *p* = 0.324; V2: t_18_ = 0.266, *p* = 0.794; V3: t_18_ = 1.186, *p* = 0.251; V4: t_18_ = 0.811, *p* = 0.428; MT+: t_18_ = 1.096, *p* = 0.288, Fig 2C, left) or Experiment 2 (V1: t_18_ = 0.751, *p* = 0.462; V2: t_18_ = 0.538, *p* = 0.597; V3: t_18_ = 0.776, *p* = 0.448; V4: t_18_ = −0.067, *p* = 0.948; MT+: t_18_ = 0.008, *p* = 0.994, Fig 2E, left). These findings confirmed that there was no difference in task difficulty or, presumably, attention between the Same and Different conditions. However, for the ignored side of the display, all of these areas showed a significantly greater response in the Same condition than that in the Different condition (Fig 2B and 2D, right), and their I_A_’s were significantly above zero in both Experiment 1 (V1: t_18_ = 4.521, *p* < 0.001; V2: t_18_ = 4.106, *p* = 0.001; V3: t_18_ = 4.661, *p* < 0.001; V4: t_18_ = 3.032, *p* = 0.007; MT+: t_18_ = 5.526, *p* < 0.001, Fig 2C, right) and Experiment 2 (V1: t_18_ = 3.961, *p* = 0.001; V2: t_18_ = 3.863, *p* = 0.001; V3: t_18_ = 3.749, *p* = 0.001; V4: t_18_ = 6.428, *p* < 0.001; MT+: t_18_ = 2.755, *p* = 0.013, Fig 2E, right). These results demonstrated that both striate and extrastriate visual areas showed the classical feature-based attention effect, with responses to the ignored stimulus significantly elevated when it shared the same feature as the attended stimulus.

To identify the area showing the largest feature-based attention effect, we submitted the I_A_ in these two experiments to a repeated-measure ANOVA with stimulus side (the target side and ignored side) and cortical area (V1–V4 and MT+) as within-participant factors. The main effect of stimulus side (Experiment 1: F_1, 18_ = 15.253, *p* = 0.001; Experiment 2: F_1, 18_ = 28.443, *p* < 0.001), the main effect of cortical area (Experiment 1: F_4, 72_ = 4.441, *p* = 0.014; Experiment 2: F_4, 72_ = 2.620, *p* = 0.042), and the interaction between these two factors (Experiment 1: F_4, 72_ = 5.173, *p* = 0.004; Experiment 2: F_4, 72_ = 5.340, *p* = 0.004) were all significant. Thus, these data were submitted to a further simple effect analysis. For all cortical areas (V1–V4 and MT+), I_A_ on the ignored side was significantly greater than that on the target side in both Experiment 1 (all t_18_ > 2.267, *p* < 0.036) and Experiment 2 (all t_18_ > 2.475, *p* < 0.024). For the target side, the main effect of cortical area was not significant in either Experiment 1 (F_4, 72_ = 0.216, *p* = 0.867) or Experiment 2 (F_4, 72_ = 0.261, *p* = 0.847). For the ignored side, however, the main effect of cortical area was significant in both Experiment 1 (F_4, 72_ = 8.199, *p* < 0.001) and Experiment 2 (F_4, 72_ = 8.059, *p* < 0.001). Post hoc paired *t* tests revealed that the I_A_ in MT+ was significantly larger than those in V1, V2, V3, and V4 (all t_18_ > 3.251, *p* < 0.044) in Experiment 1, and the I_A_ in V4 was significantly larger than those in V1, V2, V3, and MT+ (all t_18_ > 3.627, *p* < 0.019) in Experiment 2. These results indicated that the respective visual processing areas (MT+ for motion and V4 for color) showed the largest feature-based attention effect.

### Whole-brain analyses

**Fig 3:**
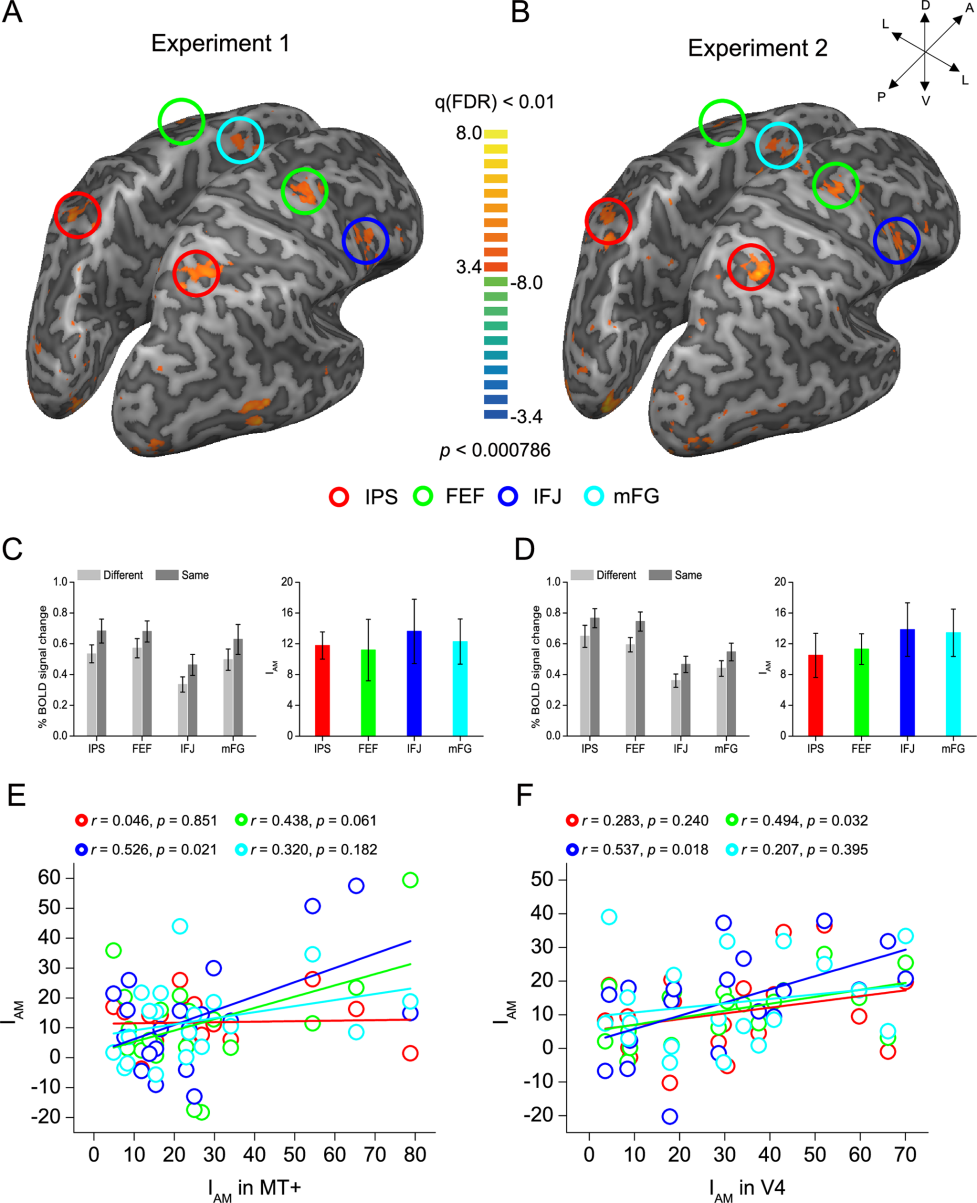
Whole-brain analysis of Experiments 1 and 2. The whole-brain search for IPS (red), FEF (green), IFJ (blue), and mFG (cyan), with all showing a significant greater response in the Same condition than the Different condition in Experiments 1 (A) and 2 (B). BOLD amplitude of these areas and their I_A_’s in Experiments 1 (C) and 2 (D). Correlations between the I_A_ in the respective visual processing areas (MT+ and V4 in Experiments 1 [E] and 2 [F], respectively) and that in these areas across individual participants. Error bars denote 1 SEM calculated across 19 participants. Data are available from the Open Science Framework (https://osf.io/8gqk6/). A, anterior; BOLD, blood oxygenation level–dependent; D, dorsal; FDR, false discovery rate; FEF, frontal eye field; I_A_, attentional modulation index; IFJ, inferior frontal junction; IPS, intraparietal sulcus; L, lateral; mFG, medial frontal gyrus; MT+, middle temporal area; P, posterior; V, ventral.

To examine potential cortical or subcortical area(s) that showed a similar feature-based attention effect to these retinotopically organized areas, we performed a group analysis and did a whole-brain search with a general linear model (GLM) procedure [47] for cortical and subcortical area(s) that showed a significant higher response in the Same condition than that in the Different condition in both Experiments 1 and 2 (note that the data from the left and right visual fields were combined). Statistical maps were thresholded at *p* < 0.01 and corrected by false discovery rate (FDR) correction [48]. The results showed that the IPS (left: −28 ± 1.12, −66 ± 1.27, 39 ± 1.19; right: 23 ± 0.93, −68 ± 1.14, 40 ± 1.78), FEF (left: −42 ± 1.21, −5 ± 0.94, 35 ± 1.28; right: 40 ±1.27, −5 ± 0.89, 39 ± 2.00), IFJ (left: −43 ± 1.62, 9 ± 1.34, 31 ± 1.98; right: 44 ± 1.51, 11 ± 1.30, 29 ± 2.04), and mFG (left: −6 ± 0.51, −2 ± 1.47, 54 ± 0.81; right: 6 ± 0.46, 2 ± 1.77, 54 ± 1.38) demonstrated a greater response in the Same condition than the Different condition in both Experiment 1 (IPS: t_18_ = 6.686, *p* < 0.001; FEF: t_18_ = 2.807, *p* = 0.012; IFJ: t_18_ = 3.253, *p* = 0.004; mFG: t_18_ = 4.186, *p* = 0.001, Fig 3C) and Experiment 2 (IPS: t_18_ = 3.650, *p* = 0.002; FEF: t_18_ = 5.652, *p* < 0.001; IFJ: t_18_ = 3.979, *p* = 0.001; mFG: t_18_ = 4.365, *p* < 0.001, Fig 3D). No significant difference of the I_A_ between Experiments 1 and 2 was found in IPS (t_18_ = 0.418, *p* = 0.681), FEF (t_18_ = −0.027, *p* = 0.979), IFJ (t_18_ = −0.039, *p* = 0.969), or mFG (t_18_ = −0.314, *p* = 0.757). Furthermore, we calculated the correlation coefficients between the I_A_ in the respective visual processing areas (MT+ and V4 in Experiments 1 and 2, respectively) and that in these cortical areas across individual participants. In Experiment 1 (Fig 3E), we found that the I_A_ in MT+ correlated significantly with that in IFJ (*r* = 0.526, *p* = 0.021) and (marginally) with that in FEF (*r* = 0.438, *p* = 0.061), but not with that in IPS (*r* = 0.046, *p* = 0.851) or mFG (*r* = 0.320, *p* = 0.182). Similarly, for Experiment 2 (Fig 3F), the I_A_ in V4 correlated significantly with that in both IFJ (r = 0.537, *p* = 0.018) and FEF (*r* = 0.494, *p* = 0.032), but not with that in IPS (*r* = 0.283, *p* = 0.240) or mFG (*r* = 0.207, *p* = 0.395). These results suggested that the spatially global effect of feature-based attention in the respective visual processing areas (MT+ for motion and V4 for color) might derive from feedback projections from FEF and/or IFJ.

### Effective connectivity analyses

**Fig 4:**
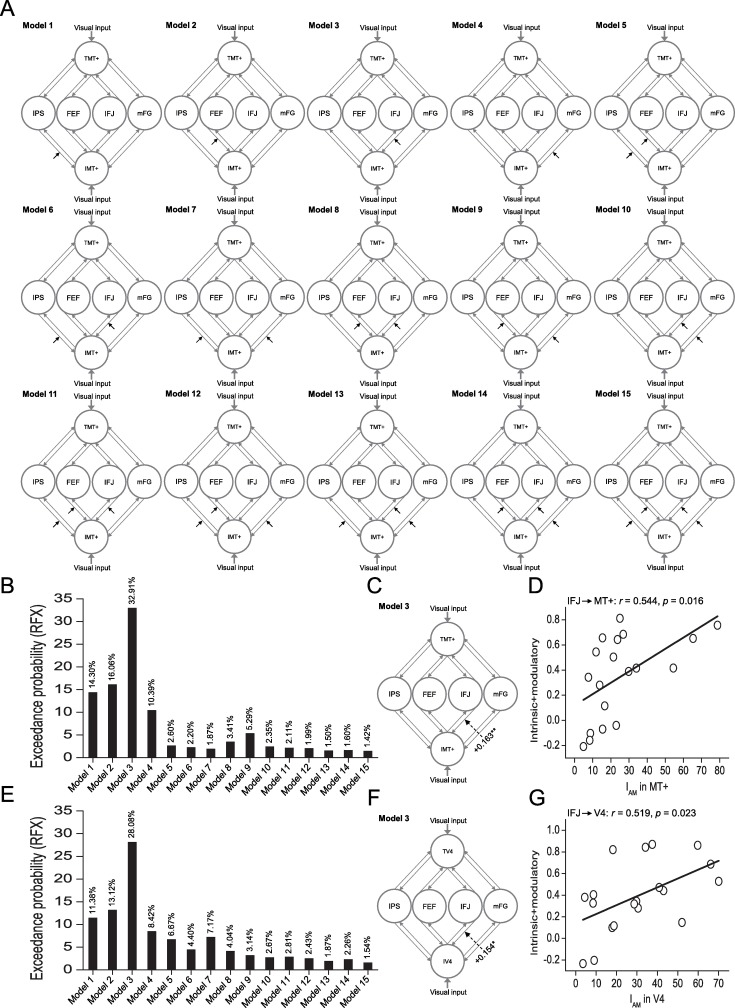
DCM of connectivities among IPS, FEF, IFJ, mFG, and the respective visual processing areas (MT+ and V4 in Experiments 1 and 2, respectively). (A) Fifteen different models used for modeling the modulatory effect of the Same condition. TMT+ and IMT+: ROI in MT+ evoked by the stimulus in the target and ignored sides, respectively; TV4 and IV4: ROI in V4 evoked by the stimulus in the target and ignored sides, respectively (F). Exceedance probabilities for the 15 models with the Same condition as the modulatory input in Experiments 1 (B) and 2 (E). The strength of the modulatory connections for the Same condition and its significance levels (*p < 0.05 and **p < 0.01, respectively) in Experiments 1 (C) and 2 (F). Correlations between the I_A_ in the respective visual processing areas (MT+ and V4 in Experiments 1 [D] and 2 [G], respectively) and effective connection strengths across individual participants. Data are available from the Open Science Framework (https://osf.io/8gqk6/). DCM, dynamic causal modeling; FEF, frontal eye field; I_A_, attentional modulation index; IFJ, inferior frontal junction; IPS, intraparietal sulcus; mFG, medial frontal gyrus; MT+, middle temporal area; RFX, random effects; ROI, region of interest.

To further examine which area is the source of the spatially global effect of feature-based attention in MT+ and V4 in Experiments 1 and 2, respectively, we used DCM analysis [49] to examine functional changes in directional connectivity among the IPS, FEF, IFJ, mFG, and the respective visual processing areas (MT+ and V4 in Experiments 1 and 2, respectively) related to the Same condition. Given the extrinsic visual input into MT+ and V4 in Experiments 1 and 2, respectively, we defined 15 different models with modulatory input (the Same condition, Fig 4A). The modulatory input could affect feedback from IPS (Model 1); from FEF (Model 2); from IFJ (Model 3); from mFG (Model 4); from both IPS and FEF (Model 5); from both IPS and IFJ (Model 6); from both IPS and mFG (Model 7); from both FEF and IFG (Model 8); from both FEF and mFG (Model 9); from both IFG and mFG (Model 10); from IPS, FEF, and IFG (Model 11); from IPS, FEF, and mFG (Model 12); from IPS, IFG, and mFG (Model 13); from FEF, IFG, and mFG (Model 14); and from all four areas (Model 15) to MT+ and V4 in Experiments 1 and 2, respectively. We examined these 15 models for modeling the modulatory effect in the Same condition for each participant.

In both Experiments 1 and 2, we computed the exceedance probability of each model [50,51]. The result showed that Model 3 was the best one to explain the modulatory effect in the Same condition in both Experiments 1 (Fig 4B) and 2 (Fig 4E). The Same condition significantly increased the feedback connectivity from IFJ to MT+ (t_18_ = 3.054, *p* = 0.007, Fig 4C) and V4 (t_18_ = 2.727, *p* = 0.014, Fig 4F) in Experiments 1 and 2, respectively. Furthermore, across individual participants, we calculated the correlation coefficients between the I_A_ in the respective visual processing areas (MT+ for motion and V4 for color) and the effective connection strengths (the sum of the intrinsic and modulatory connectivities) from IFJ to MT+ and V4 in Experiments 1 and 2, respectively. The I_A_ in MT+ and V4 correlated significantly with feedback connectivity from IFJ to MT+ (*r* = 0.544, *p* = 0.016, Fig 4D) and V4 (*r* = 0.519, *p* = 0.023, Fig 4G), respectively. Together, these results support the idea that the spatially global effect of feature-based attention in MT+ (Experiment 1) and V4 (Experiment 2) is derived by feedback from IFJ rather than from IPS, FEF, or mFG.

**Fig 5:**
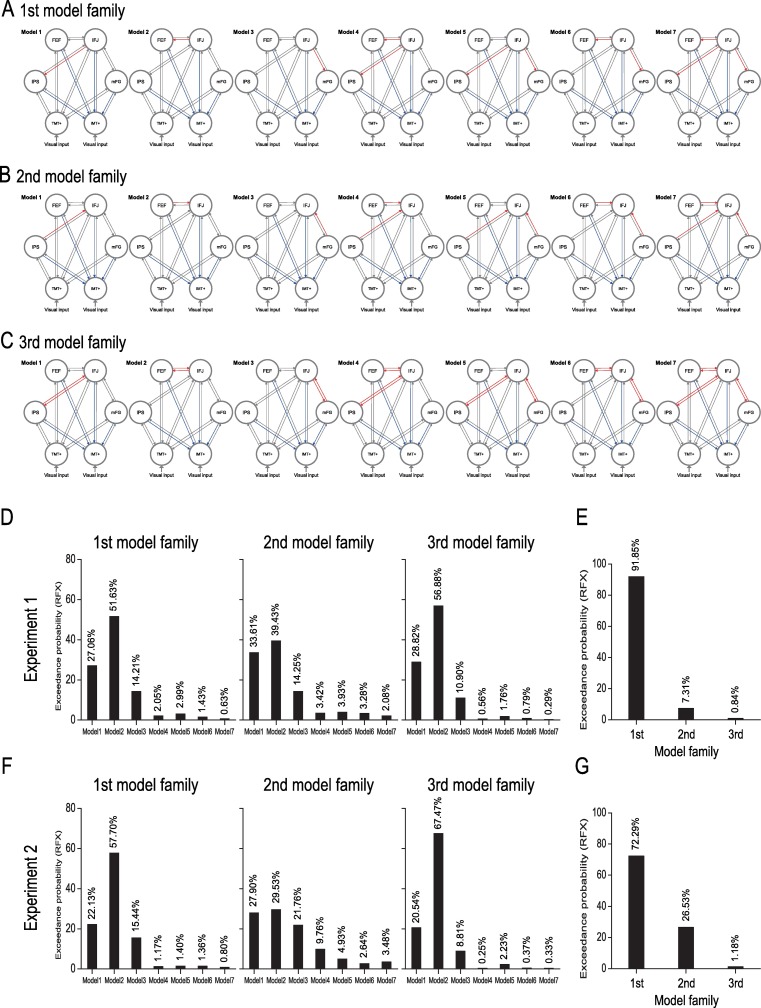
DCM of the model- and family-level analysis. (A) The first model family: seven different models used for modeling the modulatory effect of the Same condition from IFJ to the other three areas (i.e., IPS, FEF, and mFG). (B) The second model family: seven different models used for modeling the modulatory effect of the Same condition from the three areas to IFJ. (C) The third model family: the combination of the first and second model families. Exceedance probabilities for the seven models with the Same condition as the modulatory input within the three model families (model-level inference) in Experiments 1 (D) and 2 (F). Exceedance probabilities across the three model families (family-level inference) in Experiments 1 (E) and 2 (G). The red and blue lines indicate the modulatory effect of the Same condition. Data are available from the Open Science Framework (https://osf.io/8gqk6/). DCM, dynamic causal modeling; FEF, frontal eye field; IFJ, inferior frontal junction; IPS, intraparietal sulcus; mFG, medial frontal gyrus; RFX, random effects.

However, it is unclear whether the observed involvement of IFJ in the spatially global effect of feature-based attention is relayed from other areas, namely IPS, FEF, and mFG, which also showed the classical feature-based attention effect (Fig 3C and 3D). To examine this issue, we constructed three families of models with the same modulatory input (the Same condition) from IPS, IFJ, FEF, and mFG to MT+ and V4 in Experiments 1 and 2, respectively. Here, the modulatory input could affect the connection from IFJ to the other three areas (i.e., IPS, FEF, and mFG) in the first model family, or from these three areas to IFJ in the second model family, or the combination of these two families (i.e., the third model family). Specifically, in the first model family (Fig 5A), the modulatory input could affect the connection from IFJ to IPS (Model 1) to FEF (Model 2), to mFG (Model 3), to both IPS and FEF (Model 4), to both IPS and mFG (Model 5), to both FEF and mFG (Model 6), and to all three areas (Model 7). In the second model family (Fig 5B), the modulatory input could affect the connection from IPS (Model 1), from FEF (Model 2), from mFG (Model 3), from both IPS and FEF (Model 4), from both IPS and mFG (Model 5), from both FEF and mFG (Model 6), and from all three areas (Model 7) to IFJ. In the third model family (Fig 5C), each model (i.e., Models 1–7) was the combination of corresponding models from the first and second model families. We applied Bayesian model [50] comparison to select the model with the highest exceedance probability within each model family (model-level inference) and the model family with the highest exceedance probability (family-level inference). Within each model family, the results showed that Model 2 was the best one to explain the modulatory effect in the Same condition in both Experiments 1 (Fig 5D) and 2 (Fig 5F). These results confirmed our correlation analyses (Fig 3E and 3F) showing that FEF was more important than IPS and mFG in the spatially global effect of feature-based attention. More importantly, across the model families, the results showed that the first model family had a higher exceedance probability than the other two model families in both Experiments 1 (Fig 5E) and 2 (Fig 5G). These results indicate that IFJ may be the source of the spatially global effect of feature-based attention in IPS, FEF, and mFG. Furthermore, additional DCM analyses indicated that the spatially global effect in V1 was also derived by feedback from IFJ rather than by feedback from MT+ (Experiment 1) or V4 (Experiment 2); this feedback significantly predicted the spatially global effect in V1 in both experiments (S5 Fig).

Note that the contralateral and ipsilateral ROIs to the ignored side in IPS, FEF, IFJ, and mFG were pooled together in the above analyses. We next examined whether there was any difference between the contralateral and ipsilateral ROIs in mediating the spatially global effect of feature-based attention. To do so, we constructed two families of models with the contralateral ROIs only (the contralateral model family) and the ipsilateral ROIs only (the ipsilateral model family). We applied a Bayesian model [50] to compare the exceedance probability between the contralateral and ipsilateral model families. The results showed that the contralateral model family had a higher exceedance probability than the ipsilateral model family in all DCM analyses, suggesting that the contralateral ROIs were more important than the ipsilateral ROIs in the spatially global effect of feature-based attention. However, within each model family (i.e., the contralateral and ipsilateral model families), the results confirmed our previous findings by showing that: (1) the spatially global effect of feature-based attention in MT+ (Experiment 1) and V4 (Experiment 2) is derived by feedback from IFJ rather than from IPS, FEF, or mFG (S4 Fig); (2) IFJ mediates the spatially global effect of feature-based attention in IPS, FEF, and mFG and thus is considered to be a source of the spatially global effect of feature-based attention in these areas (S5 Fig); and (3) the spatially global effect of feature-based attention in V1 is dependent on feedback from IFJ rather than MT+ (Experiment 1) or V4 (Experiment 2) (S7 Fig).

In addition, we used GCM [52], a data-driven approach, to further examine which area was a potential source of the spatially global effect for feature-based attention in both experiments. In Experiment 1, for both contralateral and ipsilateral GCM analyses, our results clearly showed that the node with the highest outflow and netflow degree was node 2 (i.e., the node located in IFJ), which was thus considered to be the source of the network (S8A and S8B Fig, left and right). The node with the highest inflow degree was node 5 (i.e., the node located in MT+), which was thus considered to be the sink of the network (S8A and S8B Fig, middle). Similar results were found in Experiment 2. For both contralateral and ipsilateral GCM analyses, node 2 located in IFJ showed the highest outflow and netflow degree and thus was considered to be the source of the network (S8C and S8D Fig, left and right). Node 5 located in V4 showed the highest inflow degree and was thus considered to be the sink of the network (S8C and S8D Fig, middle). Together, our GCM results further confirmed our DCM results by showing that IFJ mediated the spatially global effect of feature-based attention in both visual processing and frontoparietal areas.

## Discussion

Numerous psychophysical [17,20,27,28], neurophysiological [22–24,29], EEG [21,30], MEG [19,31], and fMRI [18,25,32] studies have indicated the spatially global effect of feature-based attention in visual processing areas, as proposed by the “feature-similarity gain model” [23,26], whereby responses to stimuli that share features with an attended stimulus are enhanced not only at the attended location but throughout the visual field. However, how feature-based attention modulates cortical neural responses at unattended spatial locations remains unclear. Here we performed an fMRI experiment and used effective connectivity analysis to examine this issue as human participants performed motion- (Experiment 1, a speed discrimination) and color- (Experiment 2, a luminance discrimination) based attention tasks. In both experiments, our data indicated that human IFJ mediated cortical neural responses at unattended locations and could be a source of the spatially global effect for feature-based attention in the respective visual processing areas (MT+ for motion and V4 for color). First, IFJ responses showed the classic feature-based attention effect, with neural responses to the unattended stimulus significantly elevated when it shared the same feature with the attended stimulus (Fig 3C and 3D), and this response correlated significantly with the spatially global effect in MT+ (Experiment 1, Fig 3E) and V4 (Experiment 2, Fig 3F). Second, the DCM analysis indicated that the spatially global effect in MT+ (Experiment 1, Fig 4B) and V4 (Experiment 2, Fig 4E) was derived by feedback from IFJ rather than IPS, FEF, or mFG. Moreover, the increased feedback from IFJ significantly predicted the spatially global effect in MT+ (Experiment 1, Fig 4D) and V4 (Experiment 2, Fig 4G). Third, the GCM analysis indicated that, in both experiments, the node in IFJ showed the highest outflow and netflow degree and was thus considered to be the source of the network (S8 Fig). Fourth, IFJ not only mediated the spatially global effect of feature-based attention in MT+ (Experiment 1) and V4 (Experiment 2) but also in other visual processing areas (i.e., V1–V3, S3 Fig). Moreover, our additional DCM analyses also indicated that the spatially global effect in V1 was also derived by feedback from IFJ rather than by feedback from MT+ (Experiment 1) or V4 (Experiment 2), and this feedback significantly predicted the spatially global effect in V1 in both experiments (S6 Fig). Altogether, our results indicate that IFJ may be a source of the spatially global effect for feature-based attention in visual processing areas.

Our study also found the classical feature-based attention effect in IPS, FEF, and mFG, which showed a greater neural response in the Same condition than that in the Different condition (Fig 3C and 3D), consistent with previous neurophysiological [16,38–40] and brain imaging [13,41] studies showing feature-based attentional selection in the frontoparietal attention network [5,8,42,43]. Importantly, our DCM analyses suggested that the feature-based attention effect in IPS, FEF, and mFG was derived by feedback from IFJ in both of the two experiments (the first model family showed the highest exceedance probability, Fig 5). Moreover, our GCM analyses further indicated that, in both experiments, IFJ showed the highest outflow and netflow degree and was thus considered to be the source of the network containing these frontoparietal areas (S8 Fig). These results are consistent with recent neurophysiological studies [38–40]. First, Ibos and Freedman [39,40] found that neurons in the lateral intraparietal cortex (LIP, the monkey homologue of human IPS) acted as receivers of feature-based attention modulation and were not involved in the generation of top-down feature-based attention signals. Our results confirmed their findings and further indicated that the feature-based attention effect in IPS was derived by feedback from IFJ. Second, Bichot and colleagues [38] found that the ventral prearcuate (VPA) region of monkey prefrontal cortex, which could be the homologue of human IFJ [29,53], exhibited the earliest feature-based attention effects, and that inactivation of VPA impaired the animals’ ability to find targets based on their features and simultaneously eliminated the feature-based attention effect in FEF. Our results confirmed their findings by showing that the feature-based attention effect in FEF was also derived by feedback from IFJ. Additionally, both correlation (Fig 3E and 3F) and DCM analyses (Fig 5) in our study indicated that FEF was more important than IPS and mFG in feature-based attention, indicating that FEF may also be involved in the control of feature-based attention [16]. Combined with these existing neurophysiological studies [38–40], we thus speculate that IFJ may be a source of feature-based selection in both the prefrontal (i.e., FEF and mFG) and parietal (i.e., IPS) cortex.

In addition, to examine whether there was any difference between the contralateral and ipsilateral hemispheres in the spatially global effect of feature-based attention, we constructed two families of models with the contralateral ROIs only (the contralateral model family) and the ipsilateral ROIs only (the ipsilateral model family). The results showed that the contralateral model family had a higher exceedance probability than the ipsilateral model family in all DCM analyses, suggesting that the contralateral ROIs were more important than the ipsilateral ROIs in the spatially global effect of feature-based attention. However, within both the contralateral and ipsilateral model families, the results confirmed the above findings (S4 Fig and S5 Fig) and further supported the conclusion that IFJ mediates the spatially global effect of feature-based attention not only in visual processing areas but also in the frontoparietal network.

We believe that our fMRI results cannot be explained by any difference in task difficulty or, presumably, attention, between the Same and Different conditions. In Experiments 1 and 2, participants were asked to detect the speed and luminance change of the attended stimulus, respectively. Speed and luminance changes were determined by QUEST [46] before scanning to ensure that participants performed equally well for the Same and Different conditions. Additionally, our fMRI results also cannot be explained by participants inadvertently shifting their spatial attention to the ignored stimulus in the Same condition, as that would have impaired task performance [7], but there was no significant performance difference between these two conditions (S1 Fig). Note that, in our study, participants needed to attend to a specific feature on the target side for each block (Fig 1). However, we did not observe a feature-based attention effect on the target side in any area (Fig 2C and 2E) because this attended feature was the Same condition in half of the blocks and it was the Different condition in the other half of the blocks. In the Same condition, the feature, such as the red dots, on the ignored side matched the attended feature (i.e., the red dots) on the target side; a Different condition was defined as a mismatch that the feature on the ignored side was the green dots. In other words, the attended feature on the target side was always red dots between these two conditions. Thus, the BOLD response to the attended stimulus on the target side did not vary with these two conditions, and thus there was no feature-based attention effect on the target side.

The most parsimonious account of our results is identification of the IFJ as a source of the spatially global effect for feature-based attention. The IFJ is a region ventrolateral to FEF and is anatomically localized at the intersection of the precentral sulcus and the inferior frontal sulcus [54,55]. Anatomical studies have shown that IFJ has connections with sensory, parietal, and prefrontal areas [56,57], and recent resting state functional connectivity data suggest that this brain region functionally interacts with both ventral and dorsal cortical brain structures [58]. Previous studies have suggested that IFJ is involved in many different cognitive processes, including visual search [38], spatial attention [59,60], switching and Stroop tasks [61], executive control [62], working memory for maintaining and updating information [60,63–65], object-based attention induced by feature selection [66,67], and coordination of bottom-up and top-down attention [68]. Here our results extend the function of IFJ by showing a crucial involvement in the global modulation of feature-based attention. IFJ appears to control feature-based attention by actively sending top-down biasing signals for a particular feature to the visual processing areas evoked by the unattended stimulus and also to other frontoparietal areas. Notably, identifying IFJ as a source of the spatially global effect for feature-based attention derives mainly from our DCM and GCM analyses, both of which depend on time-series models of fMRI data for an interpretation of causality [69–71]. This interpretation of causality in our study finds support in previous lesion [38] and transcranial magnetic stimulation (TMS) [63,72–75] studies showing a causal effect of prefrontal cortical disruption on feature-based attention.

Furthermore, previous studies have indicated that color and motion processing engage ventral and dorsal visual processing streams, respectively. Some studies found a feature-general organization in frontoparietal cortical areas by directly comparing brain activity between attention to color and motion [41,55]. However, other studies found feature specificity in these frontoparietal cortical areas. For example, some studies found that attending to motion generally evoked larger responses than attending to color in the dorsal attention network [41,42]. Moreover, using multivariate pattern analysis (MVPA) of fMRI, some studies found attention to color and motion could evoke different patterns of activity in frontoparietal cortical areas [41]. It is important to note that, in those studies, the entire feature stimulus was located at the attended location. However, in our study, half of the feature stimulus was located at an unattended location (i.e., the ignored side of the display, see Fig 1), and we did not find this feature specificity in IPS, FEF, IFJ or mFG. Given the limitation of univariate analyses in our study, further work is needed to use MVPA of fMRI or neurophysiological techniques to address whether the spatially global effect of color-based and motion-based attentions is mediated by the same subpopulations within these frontoparietal cortical areas.

One should note that our results cannot answer a highly debated question regarding whether spatial and feature-based attention are mediated by the same or different neural mechanisms [76]. Many previous studies have suggested that the frontoparietal network is involved in the top-down control of both spatial [4,5,35–37] and feature-based attention [5,8,13,16,38–43] by mediating the neural response in visual processing areas. Our current results confirmed the role of fronto-parietal cortical areas (i.e., IPS, FEF, IFJ, and mFG) in controlling feature-based attention and identified the IFJ as a source of the spatially global effect of feature-based attention. Several studies have suggested that the IFJ’s function generalizes across both spatial and feature-based attention [60,77]. However, our study did not test the difference between spatial and feature-based attention directly and thus cannot address whether these two forms of attention are mediated by the same or different populations of IFJ neurons.

In sum, our study implicates for the first time, to the best of our knowledge, the human IFJ as the source for the spatially global effect of feature-based attention. The prominent role of the prefrontal cortex in the spatially global effect of feature-based attention evident here is consistent with recent neurophysiological and brain imaging findings that have begun to address how prefrontal areas directly top-down modulate sensory signals within posterior cortices [78] and how they covertly maintain and manipulate visual object information [79]. Combining our results with earlier studies showing a crucial involvement of IFJ in spatial [59,60], object-based [66,67], bottom-up, and top-down attention [5,68], IFJ may have a very general role in the control of attentional selection and awareness.

## Materials and methods

### Ethics statement

All participants gave written informed consent in accordance with a protocol approved by the National Institute of Mental Health (NIMH) Institutional Review Board (NIH Clinical Study Protocol 93-M-0170).

### Participants

A total of 21 adults (11 males, 19–26 years old) participated in both Experiments 1 and 2. One participant was excluded because of large head motion in the scanner (>3 mm) and another participant did not have the stamina to complete the experiments. All were naïve to the purpose of the study. They reported normal or corrected-to-normal vision and had no known neurological, psychiatric, or visual disorders.

### Stimuli

The stimulus display in both Experiments 1 and 2 was composed of two circular regions (diameter: 8.0°) in the upper visual field (centered 8.5° to the left and right of the central fixation point). One of these regions was attended (the target side) and the other was unattended (the ignored side) (Fig 1A). The target side in Experiment 1 was comprised of overlapping upward and downward moving dots (dot speed: 10.0°/second, dot diameter: 0.186°, dot luminance: approximately 76.8 cd/m^2^, dot density: 0.63/(°)^2^, each moving direction with 100% coherence), while the ignored side was a single field of dots moving either upward or downward (each moving direction with 100% coherence, Fig 1A). The target side in Experiment 2 was comprised of overlapping fields of stationary red (CIE [1931]: x = 0.620, y = 0.348) and green dots (CIE [1931]: x = 0.342, y = 0.537), while the ignored side was a single field of red or green dots (Fig 1B). In both Experiments 1 and 2, to maximally reduce the possibility that participants could focus on a single dot, half of the dots disappeared and were replaced by new dots at different random locations every 100 ms.

### fMRI experiments

Using a block design, both Experiments 1 and 2 consisted of six functional runs; three for the target side were in the left visual field (Fig 1) and the other three for the target side were in the right visual field (note that for each run, the target side was always in one hemifield and the ignored side was in the opposite hemifield). Each run consisted of eight stimulus blocks of 16 s, interleaved with eight blank intervals of 12 s. There were four different stimulus blocks: 2 (attended feature: Upward/Downward in Experiment 1; Red/Green in Experiment 2) × 2 (feature match: Same/Different). In the Same condition, the feature on the ignored side matched the attended feature on the target side (half the blocks); a Different condition was defined as a mismatch (half the blocks) (Fig 1). For example, participants attended to upward moving dots in the target side; the upward and downward moving dots in the ignored side indicate the Same and Different conditions, respectively. Each stimulus block was randomly repeated two times in each run, and the attended stimulus in each stimulus block was indicated by a colored fixation dot: red and green indicated upward moving dots and downward moving dots in Experiment 1, as well as red dots and green dots in Experiment 2, respectively. Each stimulus block consisted of eight trials; on each trial, the stimulus was presented for 0.6 s, followed by a fixed 1.4-s fixation interval, and participants did a 0.2-s speed and luminance discrimination task at threshold (75% correct, measured by QUEST [46] before scanning) in Experiments 1 and 2, respectively.

Retinotopic visual areas (V1, V2, V3, and V4) were defined by a standard phase-encoded method developed by Sereno et al. [33] and Engel et al. [34], in which participants viewed rotating wedge and expanding ring stimuli that created traveling waves of neural activity in the visual cortex. A block-design scan was used to localize the ROIs in V1–V4 and MT+ corresponding to the target and ignored stimuli (Fig 1). In both Experiments 1 and 2, the localizer scan consisted of 12 stimulus blocks of 12 s, interleaved with 12 blank intervals of 12 s. In the stimulus block, participants were asked to press one of two buttons to indicate the random luminance change (increase or decrease) of the stimulus. Whereas Experiment 1 consisted of two different stimulus blocks: stationary dots and moving dots, Experiment 2 consisted of two different stimulus blocks: gray dots and colored dots.

### MRI data acquisition

MRI data were collected using a 3T Siemens Trio scanner with a 32-channel phase-array coil. In the scanner, the stimuli were rear-projected via a video projector (refresh rate: 60 Hz; spatial resolution: 1,280×800) onto a translucent screen placed inside the scanner bore. Participants viewed the stimuli through a mirror located above their eyes. The viewing distance was 115 cm. BOLD signals were measured with an echo-planar imaging sequence (TR: 2,000 ms; TE: 30 ms; FOV: 192×192 mm^2^; matrix: 64×64; flip angle: 70; slice thickness: 3 mm; gap: 0 mm; number of slices: 34; slice orientation: axial). The bottom slice was positioned at the bottom of the temporal lobes. A 3D MPRAGE structural dataset (resolution: 1×1×1 mm^3^; TR: 2,600 ms; TE: 30 ms; FOV: 256×224 mm^2^; flip angle: 7; number of slices: 176; slice orientation: sagittal) was collected in the same session before the functional scans. Participants underwent three sessions—one for retinotopic mapping and ROI localization and the other two for Experiments 1 and 2, respectively.

### MRI data analysis

Note that the MRI data analysis, whole-brain group analysis, and DCM of this study closely followed those used by our previous studies [6, 67] and therefore, for consistency, we largely reproduce that description here, noting differences as necessary. The anatomical volume for each participant in the retinotopic mapping session was transformed into the Talairach space [80] and then inflated using BrainVoyager QX. Functional volumes in all three sessions for each participant were preprocessed, including 3D motion correction, linear trend removal, and high-pass (0.015 Hz) [81] filtering using BrainVoyager QX. Head motion within any fMRI session was <3 mm for all participants. The images were then aligned to the anatomical volume from the retinotopic mapping session and transformed into Talairach space. The first 8 s of BOLD signals were discarded to minimize transient magnetic saturation effects.

A GLM procedure was used for the ROI analysis for each participant. For each side (i.e., the target and ignored sides, Fig 1), the ROIs in V1–V3 were defined as regions that responded more strongly to the stationary gray dots than to the blank screen (*p* < 10^−3^, uncorrected). The ROI in V4 was defined as regions that responded more strongly to the stationary colored dots than to the stationary gray dots (*p* < 10^−3^, uncorrected). The ROI in MT+ was defined as regions that responded more strongly to the moving dots than to the stationary dots (*p* < 10^−3^, uncorrected). BOLD signals were extracted from these ROIs and then averaged according to the Same and Different conditions. For each stimulus block, the 2 s preceding the block served as a baseline, and the mean BOLD signal from 5 s to 16 s after stimulus onset was used as a measure of the response amplitude. For each ROI and each participant, we computed an I_A_ to quantify how much the measured response increased during the Same condition relative to the overall response to the stimuli in the ROI. The index was calculated as follows: I_A_ = (A _*Same*_ − A _*Different*_)/(A _*Same*_ + A _*Different*_)*100%, where A _*Same*_ and A _*Different*_ are the mean response amplitudes (A) in the Same and Different conditions, respectively. The index is positive whenever the mean response in the Same condition is greater than that in the Different condition.

In the whole-brain group analysis, for both Experiments 1 and 2, a fixed-effects general linear model (FFX-GLM) was performed for each participant on the spatially non-smoothed functional data in Talairach space. The design matrix consisted of two predictors (the Same and Different conditions), which were modeled as epochs using the default BrainVoyager QX`s two-gamma hemodynamic response function. Six additional parameters resulting from 3D motion correction (x, y, z rotation and translation) were included in the model. First, we calculated fixed effects analyses for each participant for the two predictors. Second, a second-level group analysis (*n* = 19) was performed with a random-effects GLM to calculate the contrast between the two predictors. Statistical maps were thresholded at *p* < 0.01 and corrected by FDR correction [48].

### DCM

To further examine which area is involved in the spatially global effect of feature-based attention in MT+ and V4 in Experiments 1 and 2, respectively, we applied DCM analysis [49] in SPM12 to our fMRI data in both experiments. For each participant and each hemisphere, using BrainVoyager QX, V4 and MT+ voxels were identified as those activated by the colored and moving dots at a significance level of *p* < 0.005, respectively; all IPS, FEF, IFJ, and mFG voxels were identified as those activated by the stimulus block at a significance level of *p* < 0.005. The mean Talairach coordinates of these voxels and the standard errors across participants for the left and right hemispheres in IPS were [−28 ± 1.12, −66 ± 1.27, 39 ± 1.19] and [23 ±0.93, −68 ± 1.14, 40 ± 1.78], respectively; those in FEF were [−42 ± 1.21, −5 ± 0.94, 35 ± 1.28] and [40 ±1.27, −5 ± 0.89, 39 ± 2.00], respectively; those in IFJ were [−42 ± 1.62, 9 ± 1.34, 31 ± 1.98] and [44 ± 1.51, 11 ± 1.30, 29 ± 2.04], respectively; and those in mFG were [−6 ± 0.51, −2 ± 1.47, 54 ± 0.81] and [6 ± 0.46, 2 ± 1.77, 54 ± 1.38], respectively. For each participant and each hemisphere, these Talairach coordinates were converted to Montreal Neurological Institute (MNI) coordinates using the tal2mni conversion utility (http://imaging.mrc-cbu.cam.ac.uk/downloads/MNI2tal/tal2mni.m). In Statistical Parametric Mapping (SPM), for each of these areas, we extracted voxels within a 4-mm sphere centered on the most significant voxel and used their time series for the DCM analysis. The estimated DCM parameters were later averaged across the two hemispheres using the Bayesian model averaging method [50].

DCMs have three sets of parameters: (1) extrinsic input into one or more regions, (2) intrinsic connectivities among the modeled regions, and (3) bilinear parameters encoding the modulations of the specified intrinsic connections by experimental manipulations [49]. The third set of parameters is used to quantify modulatory effects, which reflect increases or decreases in connectivity between two regions given some experimental manipulation, compared with the intrinsic connections between the same regions in the absence of experimental manipulation. fMRI data were modeled using GLM, with regressors for the Same condition, and a second condition comprising all visual inputs (i.e., the Same and Different conditions). This second condition was added specifically for the DCM analysis to be used as the extrinsic visual input.

Given the extrinsic visual input into MT+ and V4 in Experiments 1 and 2, respectively, we defined 15 different models with the modulatory input (the Same condition). The modulatory input could affect feedback from IPS (Model 1); from FEF (Model 2); from IFJ (Model 3); from mFG (Model 4); from both IPS and FEF (Model 5); from both IPS and IFJ (Model 6); from both IPS and mFG (Model 7); from both FEF and IFG (Model 8); from both FEF and mFG (Model 9); from both IFG and mFG (Model 10); from IPS, FEF, and IFG (Model 11); from IPS, FEF, and mFG (Model 12), from IPS, IFG, and mFG (Model 13); from FEF, IFG, and mFG (Model 14); and from all four areas (Model 15) to MT+ and V4 in Experiments 1 and 2, respectively (Fig 4A). We examined these 15 models for modeling the modulatory effect by the Same condition and fit each of these 15 models for each participant. Using a hierarchical Bayesian approach [50], we compared the 15 models by computing the exceedance probability of each model, i.e., the probability to which a given model is more likely than any other included model to have generated data from a randomly selected participant. In the best model (Model 3), we examined the modulatory effect by the Same condition.

Moreover, to examine whether the observed involvement of IFJ in the spatially global effect of feature-based attention is relayed from the other three areas, namely IPS, FEF, and mFG, we constructed three families of models with the same modulatory input (the Same condition) from IPS, IFJ, FEF, and mFG to MT+ and V4 in Experiments 1 and 2, respectively. Note that the modulatory input could affect the connection from IFJ to the other three areas (i.e., IPS, FEF, and mFG) in the first model family, or from these three areas to IFJ in the second model family, or the combination of these two families (i.e., the third model family). Specifically, in the first model family (Fig 5A), the modulatory input could affect the connection from IFJ to IPS (Model 1), to FEF (Model 2), to mFG (Model 3), to both IPS and FEF (Model 4), to both IPS and mFG (Model 5), to both FEF and mFG (Model 6), and to all three areas (Model 7). In the second model family (Fig 5B), the modulatory input could affect the connection from IPS (Model 1), from FEF (Model 2), from mFG (Model 3), from both IPS and FEF (Model 4), from both IPS and mFG (Model 5), from both FEF and mFG (Model 6), and from all three areas (Model 7) to IFJ. In the third model family (Fig 5C), each model (i.e., Models 1–7) was the combination of corresponding models from the first and second model families. We applied Bayesian model [50] comparison to select the model with the highest exceedance probability within each model family (model-level inference) and the model family with the highest exceedance probability (family-level inference).

### Eye movement recording

Eye movements were recorded with an EyeLink 1000 Plus system (SR Research, Ltd., Mississauga, Ontario, Canada) in a psychophysics lab (the scanner did not have an applicable eye tracking system). Recording (500 Hz) was performed when participants performed the same task as Experiments 1 and 2. S2 Fig shows that participants’ eye movements were small and statistically indistinguishable between the Same and Different conditions.

## Supporting information

S1 Fig Change detection thresholds, response accuracies, and reaction times for the Same and Different conditions in Experiments 1 and 2. In Experiment 1, the speed change detection thresholds (mean degree ± SEM) were 3.169 ± 0.285, 3.285 ± 0.301, 3.234 ± 0.306, and 3.477 ± 0.322 (A); the accuracy rates (mean percent correct ± SEM) were 73.355 ± 2.448%, 73.432 ± 1.987%, 71.711 ± 1.942%, and 72.840 ± 2.306%; and the reaction times (mean reaction time ± SEM) were 444.763 ± 18.715 ms, 451.345 ± 18.941 ms, 442.994 ± 18.815 ms, and 445.724 ± 19.264 ms for Upward-Different, Upward-Same, Downward-Different, and Downward-Same conditions, respectively (C). Paired *t* tests revealed that there was no significant difference (all *p* > 0.05) in all these measurements between the Same and Different conditions in Experiment 1. In Experiment 2, the luminance change detection thresholds (B) were 7.698 ± 0.493, 7.798 ± 0.505, 13.950 ± 1.447, and 13.500 ± 1.438; the accuracy rates were 73.454 ± 2.094%, 71.601 ± 1.838%, 72.127 ± 1.340%, and 71.557 ± 1.623%; and the reaction times were 423.762 ± 20.368 ms, 424.367 ± 22.839 ms, 435.620 ± 20.860 ms, and 428.500 ± 23.007 ms for Red-Different, Red-Same, Green-Different, and Green-Same conditions, respectively (D). Paired *t* tests revealed that there was no significant difference (all *p* > 0.05) in all these measurements between the Same and Different conditions in Experiment 2. Error bars denote 1 SEM calculated across 19 participants. Data are available from the Open Science Framework (https://osf.io/8gqk6/).
(TIF)

S2 Fig Eye movement data for the Same and Different conditions in Experiments 1 and 2. Horizontal and vertical eye positions after removing blinks and artifacts for the Same (bottom) and Different (top) conditions in Experiments 1 (left) and 2 (right). Eye movements were small and eye position distributions were very similar between the Same and Different conditions. *t* tests showed that the horizontal and vertical mean eye positions of all the distributions did not deviate significantly from the fixation point (all *p* > 0.05). Data are available from the Open Science Framework (https://osf.io/8gqk6/).
(TIF)

S3 Fig Correlations between the IA in IFJ and that in other visual processing areas (V1–V4 in Experiment 1; V1–V3 and MT+ in Experiment 2) across individual participants. We calculated the correlation coefficients between the I_A_ in IFJ and that in other visual processing areas across individual participants. In Experiment 1 (A), we found that the I_A_ in IFJ correlated significantly with that in V1 (*r* = 0.574, *p* = 0.010), V2 (*r* = 0.490, *p* = 0.033), and (marginally) V3 (*r* = 0.434, *p* = 0.063), but not with that in V4 (*r* = 0.217, *p* = 0.372). In Experiment 2 (B), the I_A_ in IFJ correlated significantly with that in V1 (*r* = 0.509, *p* = 0.026), V2 (*r* = 0.509, *p* = 0.026), and (marginally) V3 (*r* = 0.430, *p* = 0.066), but not with that in MT+ (*r* = 0.204, *p* = 0.403). These correlation analyses suggest that the spatially global effect of feature-based attention in V1–V3 may derive from feedback projections from IFJ in both experiments. Data are available from the Open Science Framework (https://osf.io/8gqk6/). I_A_, attentional modulation index; IFJ, inferior frontal junction; MT+, middle temporal area; V1, primary visual cortex.
(TIF)

S4 Fig DCM of the model- and family-level analysis among IPS, FEF, IFJ, mFG, and the respective visual processing areas (MT+ and V4) in Experiments 1 and 2, respectively. (A) The contralateral model family: each model (i.e., Models 1–15) was the corresponding model from Fig 4 with IPS, FEF, IFJ, and mFG in the hemisphere contralateral to the ignored side. CIPS, CFEF, CIFJ, and CmFG: ROI of contralateral IPS, FEF, IFJ, and mFG to the ignored side, respectively. (B) The ipsilateral model family: each model with IPS, FEF, IFJ, and mFG in the hemisphere ipsilateral to the ignored side. IIPS, IFEF, IIFJ, and ImFG: ROI of ipsilateral IPS, FEF, IFJ, and mFG to the ignored side, respectively. We applied a Bayesian model [50] comparison to select the model with the highest exceedance probability within each model family (model-level inference) and the model family with the highest exceedance probability (family-level inference). Within each model family (i.e., the contralateral and ipsilateral model families), the results showed that Model 3 was the best one to explain the modulatory effect in the Same condition in both Experiment 1 (exceedance probabilities of Models 1–15, the contralateral model family: 13.70%, 12.96%, 32.64%, 7.65%, 2.05%, 2.08%, 2.07%, 2.57%, 2.37%, 4.53%, 1.70%, 1.76%, 2.11%, 3.06%, and 8.75%, respectively; the ipsilateral model family: 14.54%, 20.11%, 27.66%, 10.36%, 2.66%, 2.34%, 2.14%, 3.49%, 5.40%, 2.53%, 2.00%, 1.74%, 1.87%, 1.63%, and 1.53%, respectively [C]) and Experiment 2 (exceedance probabilities of Models 1–15, the contralateral model family: 10.65%, 12.69%, 28.37%, 4.77%, 7.43%, 4.13%, 11.76%, 4.13%, 2.75%, 2.62%, 2.44%, 2.54%, 1.79%, 2.41%, and 1.52%, respectively; the ipsilateral model family: 8.77%, 18.33%, 21.39%, 11.11%, 2.90%, 4.68%, 5.75%, 4.41%, 4.29%, 4.62%, 3.03%, 2.86%, 2.68%, 2.63%, and 2.55%, respectively, [E]). These results further confirmed our results that the spatially global effect of feature-based attention in MT+ (Experiment 1) and V4 (Experiment 2) was derived by feedback from IFJ rather than from IPS, FEF, or mFG. Moreover, we found that the contralateral model family had a higher exceedance probability than the ipsilateral model family in both Experiment 1 (exceedance probability, the contralateral model family: 71.75%; the ipsilateral model family: 28.25% [D]) and Experiment 2 (exceedance probability, the contralateral model family: 74.35%; the ipsilateral model family: 25.65% [F]). These results indicate a more crucial role of feedback from the contralateral IFJ than the ipsilateral IFJ in the spatially global effect of feature-based attention in MT+ (Experiment 1) and V4 (Experiment 2). Data are available from the Open Science Framework (https://osf.io/8gqk6/). DCM, dynamic causal modeling; FEF, frontal eye field; IFJ, inferior frontal junction; IPS, intraparietal sulcus; mFG, medial frontal gyrus; MT+, middle temporal area; ROI, region of interest.
(TIF)

S5 Fig DCM of the contralateral and ipsilateral ROIs for Fig 5. (A) The contralateral model family: each model (i.e., Models 1–7) and sub-model family (i.e., the first, second, and third model families) was the corresponding model and model family from Fig 5 with IPS, FEF, IFJ, and mFG in the hemisphere contralateral to the ignored side. CIPS, CFEF, CIFJ, and CmFG: ROI of contralateral IPS, FEF, IFJ, and mFG to the ignored side, respectively. (B) The ipsilateral model family: each model and sub-model family with IPS, FEF, IFJ, and mFG in the hemisphere ipsilateral to the ignored side. IIPS, IFEF, IIFJ, and ImFG: ROI of ipsilateral IPS, FEF, IFJ, and mFG to the ignored side, respectively. We applied a Bayesian model [50] comparison to select the model with the highest exceedance probability within each sub-model family (model-level inference) and the sub-model family with the highest exceedance probability (sub-family-level inference). We also applied a Bayesian model to compare the exceedance probability between the contralateral and ipsilateral model families. For the contralateral ROIs, within each sub-model family, the results showed that Model 2 was the best one to explain the modulatory effect in the Same condition in both Experiment 1 (exceedance probabilities of Models 1–7, the first model family: 20.23%, 47.26%, 17.34%, 1.94%, 9.39%, 2.71%, and 1.13%, respectively; the second model family: 35.24%, 39.28%, 11.89%, 4.67%, 2.61%, 3.61%, and 2.70%, respectively; the third model family: 34.32%, 55.19%, 8.40%, 0.58%, 0.61%, 0.65%, and 0.25%, respectively [C], left) and Experiment 2 (exceedance probabilities of Models 1–7, the first model family: 18.96%, 52.06%, 10.73%, 2.38%, 12.31%, 2.42%, and 1.14%, respectively; the second model family: 11.73%, 65.08%, 6.98%, 3.22%, 6.19%, 2.25%, and 4.55%, respectively; the third model family: 20.88%, 78.18%, 0.75%, 0.06%, 0.06%, 0.05%, and 0.02%, respectively [F], left). Similar results were found for the ipsilateral ROIs; within each sub-model family, Model 2 was the best one to explain the modulatory effect in the Same condition in both Experiment 1 (exceedance probabilities of Models 1–7, the first model family: 28.67%, 49.79%, 13.77%, 2.49%, 3.09%, 1.43%, and 0.76%, respectively; the second model family: 33.35%, 41.56%, 12.81%, 3.46%, 3.05%, 3.51%, and 2.26%, respectively; the third model family: 38.35%, 44.56%, 13.26%, 0.77%, 1.76%, 0.70%, and 0.60%, respectively [C], right) and Experiment 2 (exceedance probabilities of Models 1–7, the first model family: 23.17%, 56.25%, 15.49%, 1.32%, 1.50%, 1.62%, and 0.65%, respectively; the second model family: 26.30%, 29.48%, 10.12%, 22.49%, 5.65%, 2.57%, and 3.39%, respectively; the third model family: 19.05%, 68.13%, 9.74%, 0.27%, 2.04%, 0.43%, and 0.34%, respectively [F], right). These results confirmed our results showing that FEF was more important than IPS and mFG in the spatially global effect of feature-based attention. More importantly, for both contralateral and ipsilateral ROIs, across the sub-model families, the results showed that the first model family had a higher exceedance probability than the other two model families in both Experiment 1 (the contralateral ROIs: exceedance probabilities of sub-model families 1–3: 91.83%, 6.94%, and 1.23%, respectively; the ipsilateral ROIs: exceedance probabilities of sub-model families 1–3: 76.83%, 22.64%, and 0.53%, respectively [D]) and Experiment 2 (the contralateral ROIs: exceedance probabilities of sub-model families 1–3: 56.54%, 40.95%, and 2.51%, respectively; the ipsilateral ROIs: exceedance probabilities of sub-model families 1–3: 54.76%, 44.13%, and 1.11%, respectively [G]). These results confirm that IFJ may be the source of the spatially global effect of feature-based attention in IPS, FEF, and mFG. Besides, we found that the contralateral model family had a higher exceedance probability than the ipsilateral model family in both Experiment 1 (exceedance probability, the contralateral model family: 82.66%; the ipsilateral model family: 17.34% [E]) and Experiment 2 (exceedance probability, the contralateral model family: 74.74%; the ipsilateral model family: 25.26% [H]). These results indicate that the contralateral IFJ may be more important than the ipsilateral IFJ in mediating the spatially global effect of feature-based attention in IPS, FEF, and mFG. Data are available from the Open Science Framework (https://osf.io/8gqk6/). DCM, dynamic causal modeling; FEF, frontal eye field; IFJ, inferior frontal junction; IPS, intraparietal sulcus; mFG, medial frontal gyrus; ROI, region of interest.
(TIF)

S6 Fig DCM of connectivities among IFJ, V1, and the respective visual processing areas (MT+ and V4) in Experiments 1 and 2, respectively. We examined whether the spatially global effect of feature-based attention in V1 is dependent on feedback from IFJ or from MT+ and V4 in Experiments 1 and 2, respectively. To examine this issue, we further defined three different models with the modulatory input (the Same condition [A]). The modulatory input could affect the feedback to V1 from IFJ (Model 1), from MT+ and V4 in Experiments 1 and 2, respectively (Model 2), or from all these areas (Model 3). TV1 and IV1: ROI in V1 evoked by the stimulus in the target and ignored sides, respectively. We examined these three models for the modulatory effect by the Same condition and fit each of the three models for each participant. Using a hierarchical Bayesian approach, we compared the three models by computing the exceedance probability of each model. In the best model, we examined the modulatory effect by the Same condition. The result showed that Model 1 was the best one to explain the modulatory effect in the Same condition in both Experiment 1 (exceedance probability, Model 1: 62.50%; Model 2: 25.43%; Model 3: 12.07% [B]) and Experiment 2 (exceedance probability, Model 1: 56.99%; Model 2: 27.43%; Model 3: 15.58% [E]). The Same condition significantly increased the feedback connectivity from IFJ to V1 in both Experiment 1 (t_18_ = 2.703, *p* = 0.015 [C]) and Experiment 2 (t_18_ = 2.252, *p* = 0.037 [F]) (*p < 0.05). Across individual participants, the feedback connectivity from IFJ to V1 correlated significantly with the I_A_ in V1 in both Experiment 1 (*r* = 0.511, *p* = 0.025 [D]) and Experiment 2 (*r* = 0.463, *p* = 0.046 [G]). These results indicate that the spatially global effect of feature-based attention in V1 is dependent on feedback from IFJ rather than MT+ (Experiment 1) or V4 (Experiment 2). Data are available from the Open Science Framework (https://osf.io/8gqk6/). DCM, dynamic causal modeling; I_A_, attentional modulation index; IFJ, inferior frontal junction; MT+, middle temporal area; ROI, region of interest; V1, primary visual cortex.
(TIF)

S7 Fig DCM of the model- and family-level analysis among IFJ, V1, and the respective visual processing areas (MT+ and V4) in Experiments 1 and 2, respectively. (A) The contralateral model family: each model (i.e., Models 1–3) was the corresponding model from S6 Fig with IFJ in the hemisphere contralateral to the ignored side. CIFJ: the ROI of contralateral IFJ to the ignored side. (B) The ipsilateral model family: each model with IFJ in the hemisphere ipsilateral to the ignored side. IIFJ: the ROI of ipsilateral IFJ to the ignored side. We applied a Bayesian model [50] comparison to select the model with the highest exceedance probability within each model family (model-level inference) and the model family with the highest exceedance probability (family-level inference). Within each model family (i.e., the contralateral and ipsilateral model families), the results showed that Model 1 was the best one to explain the modulatory effect in the Same condition in both Experiment 1 (exceedance probabilities of Models 1–3, the contralateral model family: 73.07%, 14.13%, and 12.80%, respectively; the ipsilateral model family: 59.72%, 28.77%, and 11.51%, respectively [C]) and Experiment 2 (exceedance probabilities of Models 1–3, the contralateral model family: 56.57%, 24.56%, and 18.87%, respectively; the ipsilateral model family: 50.61%, 35.31%, and 14.08%, respectively [E]). These results further confirmed our results that the spatially global effect of feature-based attention in V1 is dependent on feedback from IFJ rather than MT+ (Experiment 1) or V4 (Experiment 2). Moreover, we found that the contralateral model family had a higher exceedance probability than the ipsilateral model family in both Experiment 1 (exceedance probability, the contralateral model family: 74.24%; the ipsilateral model family: 25.76% [D]) and Experiment 2 (exceedance probability, the contralateral model family: 82.64%; the ipsilateral model family: 17.36% [F]). These results indicate a more crucial role of feedback from the contralateral IFJ than the ipsilateral IFJ in the spatially global effect of feature-based attention in V1 in both experiments. Data are available from the Open Science Framework (https://osf.io/8gqk6/). DCM, dynamic causal modeling; IFJ, inferior frontal junction; MT+, middle temporal area; ROI, region of interest; V1, primary visual cortex.
(TIF)

S8 Fig Results of GCM. It is well known that a general concern about DCM is the problem of its hypothesis-driven approach. Thus, we used GCM [52], a data-driven approach, to further examine which area was a potential source of the spatially global effect for feature-based attention in both experiments. Unlike DCM, GCM does not require any a priori prespecification about the connectivity structure, because it studies the temporal precedence among fMRI time series using the concept of Granger causality [52], and it has been applied in numerous brain imaging studies regarding the effective connectivity analysis [82–88]. In our study, we used the GMAC toolbox (http://selene.bioing.polimi.it/BBBlab/GMAC), BIOSIG toolbox (http://biosig.sourceforge.net/), and MARSeille Boîte À Région d’Intérêt (MarsBaR) toolbox (http://marsbar.sourceforge.net/) [89] in SPM12 to our fMRI data in both experiments. First, nodes definition: all the nodes of the network were the active clusters from a group GLM analysis that showed a higher response in the Same condition than the Different condition at a significance level of *p* < 0.05 (corrected by FDR correction [48]) in two experiments. In Experiment 1, the MNI coordinates of the first cluster in the left and right hemispheres were [−38.4, −4.89, 38.7] and [42.1, −1.49, 39.8], respectively; those of the second were [−29.9, 25.2, 28.4] and [36.8, 25.9, 31.2], respectively; those of the third were [−28.6, −64.8, 32] and [26.9, −68.6, 36.9], respectively; those of the fourth were [−6.04, −0.326, 54.1] and [2.09, 3.81, 54.2], respectively; and those of the fifth were [−40, −70, −6.11] and [39, −68.9, −8.69], respectively. The first to fifth clusters were localized in bilateral FEF, IFJ, IPS, mFG, and MT+, respectively, and they were defined as Nodes 1 through 5, respectively (A and B). In Experiment 2, the MNI coordinates of the first cluster in the left and right hemispheres were [−40.6, −3.50, 38.7] and [42.2, 0.317, 41.2], respectively; those of the second were [−31, 25.6, 30.4] and [39.8, 26.3, 30.8], respectively; those of the third were [−26.3, −65.2, 38] and [27.9, −64.1, 40.7], respectively; those of the fourth were [−6.16, 1.84, 57.7] and [10.3, 4.84, 57.1], respectively; and those of the fifth were [−26.4, −75.1, −21.1] and [22.2, −72.7, −18.1], respectively. The first to fifth clusters were localized in bilateral FEF, IFJ, IPS, mFG, and V4, respectively, and they were defined as Nodes 1 through 5, respectively (C and D). All coordinates of these clusters were saved with the SPM MarsBaR toolbox (http://marsbar.sourceforge.net/). Second, extraction of fMRI time series: for each of these clusters and each participant, we used GMAC toolbox (http://selene.bioing.polimi.it/BBBlab/GMAC) to extract the preprocessed BOLD time series for GCM analysis after removal of the nuisance variables (i.e., the remaining sources of spurious variance [89]) and session-specific grand mean scaling (the mean value of the intracerebral voxels over space and time was set as 100 [90]). The significance of Granger causal measures in our study was assessed using the Bootstrapping (10,000 times) surrogation method [81] and FDR correction (*p* < 0.05) [48]. (A) GCM analysis with IPS, FEF, IFJ, and mFG in the hemisphere contralateral to the ignored side in Experiment 1. CIPS, CFEF, CIFJ, and CmFG: node in contralateral IPS, FEF, IFJ, and mFG to the ignored side, respectively. (B) GCM analysis with IPS, FEF, IFJ, and mFG in the hemisphere ipsilateral to the ignored side in Experiment 1. IIPS, IFEF, IIFJ, and ImFG: node in ipsilateral IPS, FEF, IFJ, and mFG to the ignored side, respectively. IMT+: the node localized in MT+ was evoked by the stimulus in the ignored side. (C and D) GCM analyses with contralateral and ipsilateral nodes in Experiment 2. IV4: the node localized in V4 was evoked by the stimulus in the ignored side. Note that both color and thickness of lines indicate the Granger causal strength. In Experiment 1, for both contralateral and ipsilateral GCM analyses, our results clearly showed that the node with the highest outflow and netflow degree was Node 2 (i.e., the node located in IFJ, and heighted by a dashed circle), which was thus considered to be the source of the network (A and B, left and right). The node with the highest inflow degree was Node 5 (i.e., the node located in MT+, and heighted by a dashed circle), which was thus considered to be the sink of the network (A and B, middle). Similar results were found in Experiment 2. For both contralateral and ipsilateral GCM analyses, Node 2, located in IFJ (heighted by a dashed circle), showed the highest outflow and netflow degree and was thus considered to be the source of the network (C and D, left and right). Node 5, located in V4 (heighted by a dashed circle), showed the highest inflow degree and was thus considered to be the sink of the network (C and D, middle). Together, our GCM results further confirmed our DCM results by showing that IFJ mediated the spatially global effect of feature-based attention in both visual processing and frontoparietal areas. Data are available from the Open Science Framework (https://osf.io/8gqk6/). BOLD, blood oxygenation level–dependent; DCM, dynamic causal modeling; FDR, false discovery rate; FEF, frontal eye field; fMRI, functional magnetic resonance imaging; GCM, Granger causality modeling; GLM, general linear model; GMAC, Granger multivariate autoregressive connectivity; IFJ, inferior frontal junction; IPS, intraparietal sulcus; MarsBaR, MARSeille Boîte À Région d’Intérêt; mFG, medial frontal gyrus; MNI, Montreal Neurological Institute; MT+, middle temporal area; SPM, Statistical Parametric Mapping.
(TIF)

## Abbreviations

BOLD: blood oxygenation level–dependent
DCM: dynamic causal modeling
EEG: electroencephalographic
FDR: false discovery rate
FEF: frontal eye field
FFX-GLM: fixed-effects general linear model
fMRI: functional magnetic resonance imaging
GCM: Granger causality modeling
GLM: general linear model
GMAC: Granger multivariate autoregressive connectivity
I_A_: attentional modulation index
IFJ: inferior frontal junction
IPS: intraparietal sulcus
LIP: lateral intraparietal cortex
MarsBaR: MARSeille Boîte À Région d’Intérêt
MEG: magnetoencephalogram
mFG: medial frontal gyrus
MNI: Montreal Neurological Institute
MT+: middle temporal area
MVPA: multivariate pattern analysis
NIMH: National Institute of Mental Health
RFX: random effects
ROI: region of interest
SPM: Statistical Parametric Mapping
TMS: transcranial magnetic stimulation
VPA: ventral prearcuate
V1: primary visual cortex/striate visual cortex
