# Supplementary figures and images for: The role of inferior frontal junction in controlling the spatially global effect of feature-based attention in human visual areas
Source: PLoS Biol. 2018 Jun 25;16(6):e2005399. doi: 10.1371/journal.pbio.2005399 (PMC6034892; doi:10.1371/journal.pbio.2005399)

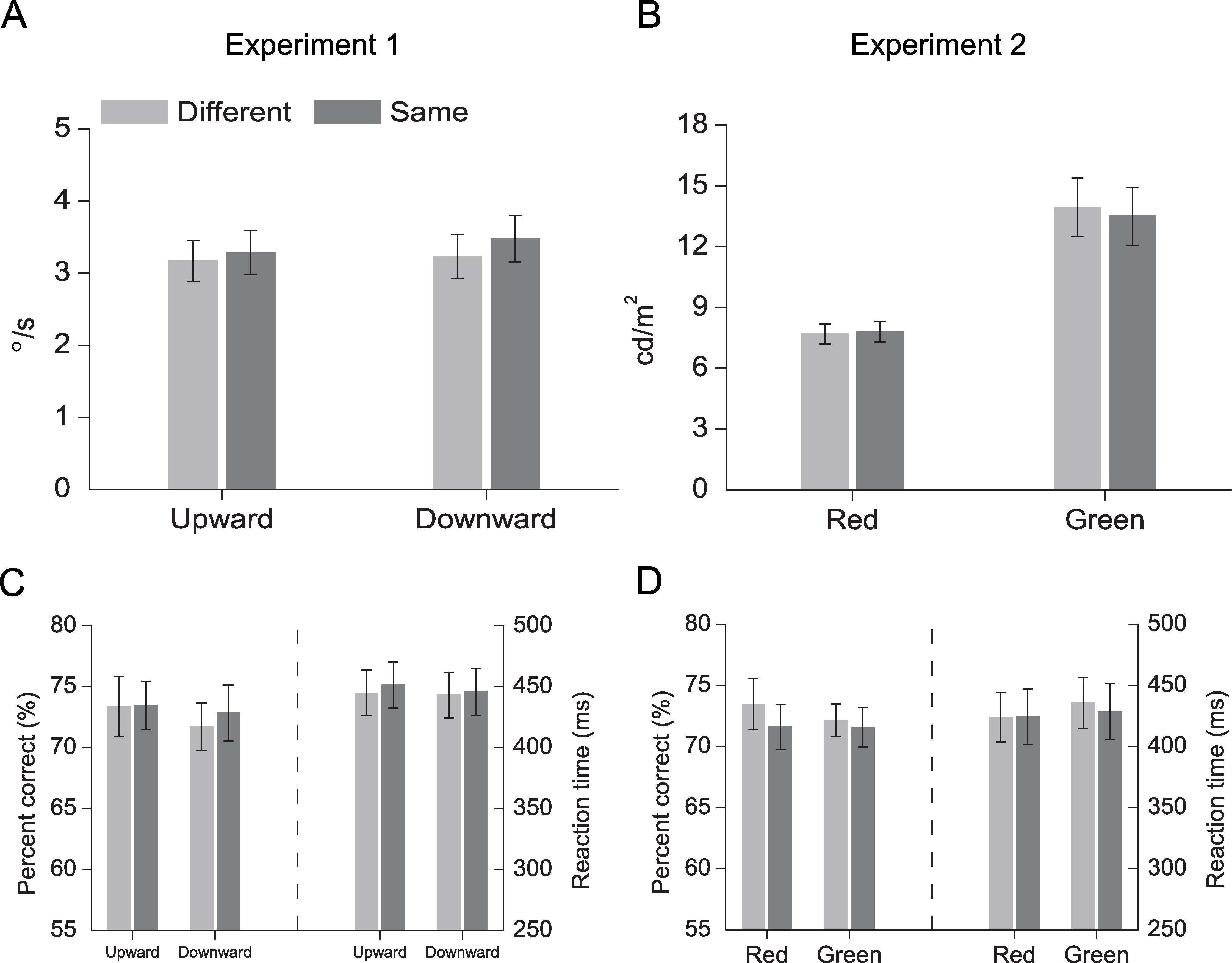

Supplement: S1 Fig — In Experiment 1, the speed change detection thresholds (mean degree ± SEM) were 3.169 ± 0.285, 3.285 ± 0.301, 3.234 ± 0.306, and 3.477 ± 0.322 (A); the accuracy rates (mean percent correct ± SEM) were 73.355 ± 2.448%, 73.432 ± 1.987%, 71.711 ± 1.942%, and 72.840 ± 2.306%; and the reaction times (mean reaction time ± SEM) were 444.763 ± 18.715 ms, 451.345 ± 18.941 ms, 442.994 ± 18.815 ms, and 445.724 ± 19.264 ms for Upward-Different, Upward-Same, Downward-Different, and Downward-Same conditions, respectively (C). Paired t tests revealed that there was no significant difference (all p > 0.05) in all these measurements between the Same and Different conditions in Experiment 1. In Experiment 2, the luminance change detection thresholds (B) were 7.698 ± 0.493, 7.798 ± 0.505, 13.950 ± 1.447, and 13.500 ± 1.438; the accuracy rates were 73.454 ± 2.094%, 71.601 ± 1.838%, 72.127 ± 1.340%, and 71.557 ± 1.623%; and the reaction times were 423.762 ± 20.368 ms, 424.367 ± 22.839 ms, 435.620 ± 20.860 ms, and 428.500 ± 23.007 ms for Red-Different, Red-Same, Green-Different, and Green-Same conditions, respectively (D). Paired t tests revealed that there was no significant difference (all p > 0.05) in all these measurements between the Same and Different conditions in Experiment 2. Error bars denote 1 SEM calculated across 19 participants. Data are available from the Open Science Framework (https://osf.io/8gqk6/). (TIF) [file pbio.2005399.s001.tif]

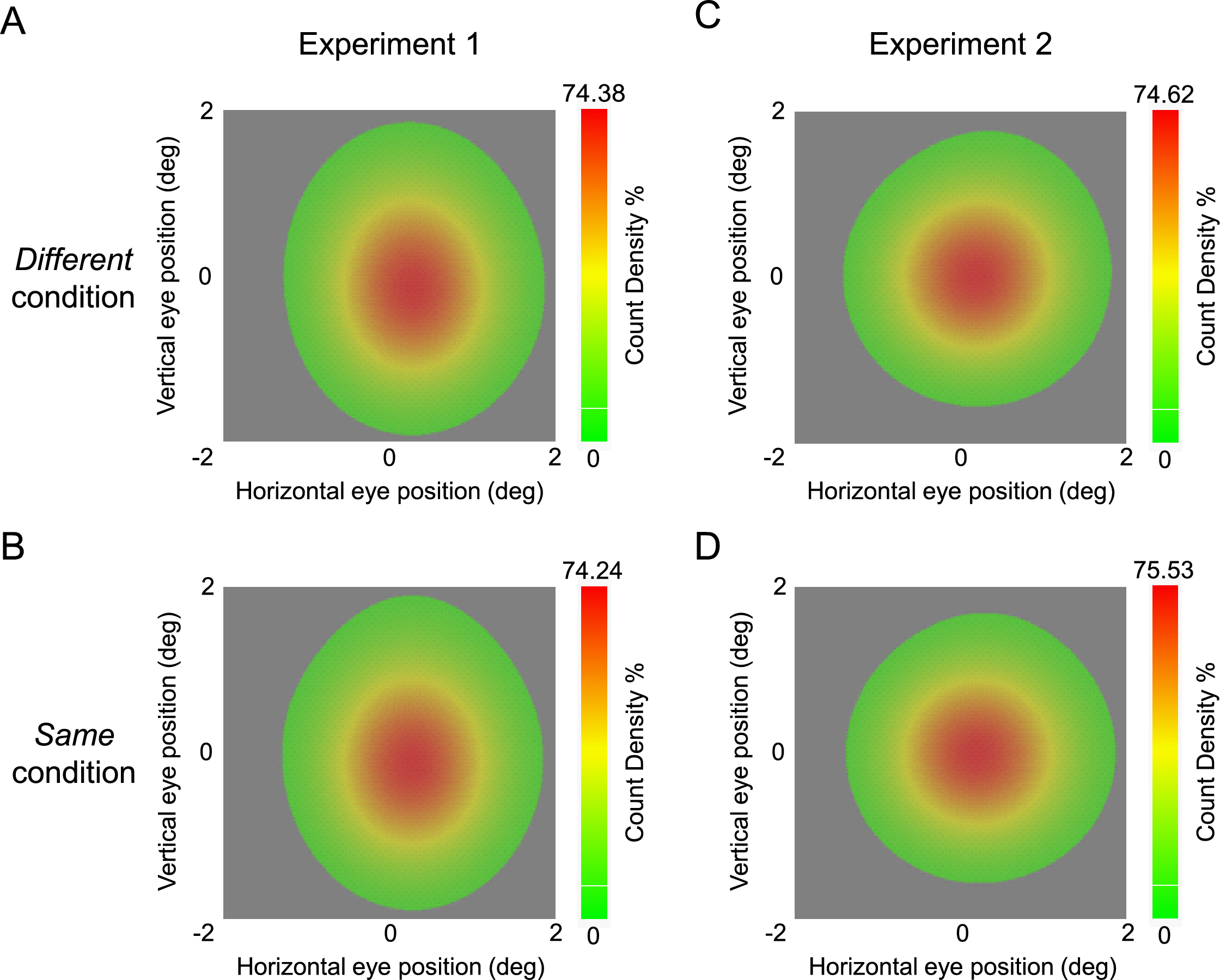

Supplement: S2 Fig — Horizontal and vertical eye positions after removing blinks and artifacts for the Same (bottom) and Different (top) conditions in Experiments 1 (left) and 2 (right). Eye movements were small and eye position distributions were very similar between the Same and Different conditions. t tests showed that the horizontal and vertical mean eye positions of all the distributions did not deviate significantly from the fixation point (all p > 0.05). Data are available from the Open Science Framework (https://osf.io/8gqk6/). (TIF) [file pbio.2005399.s002.tif]

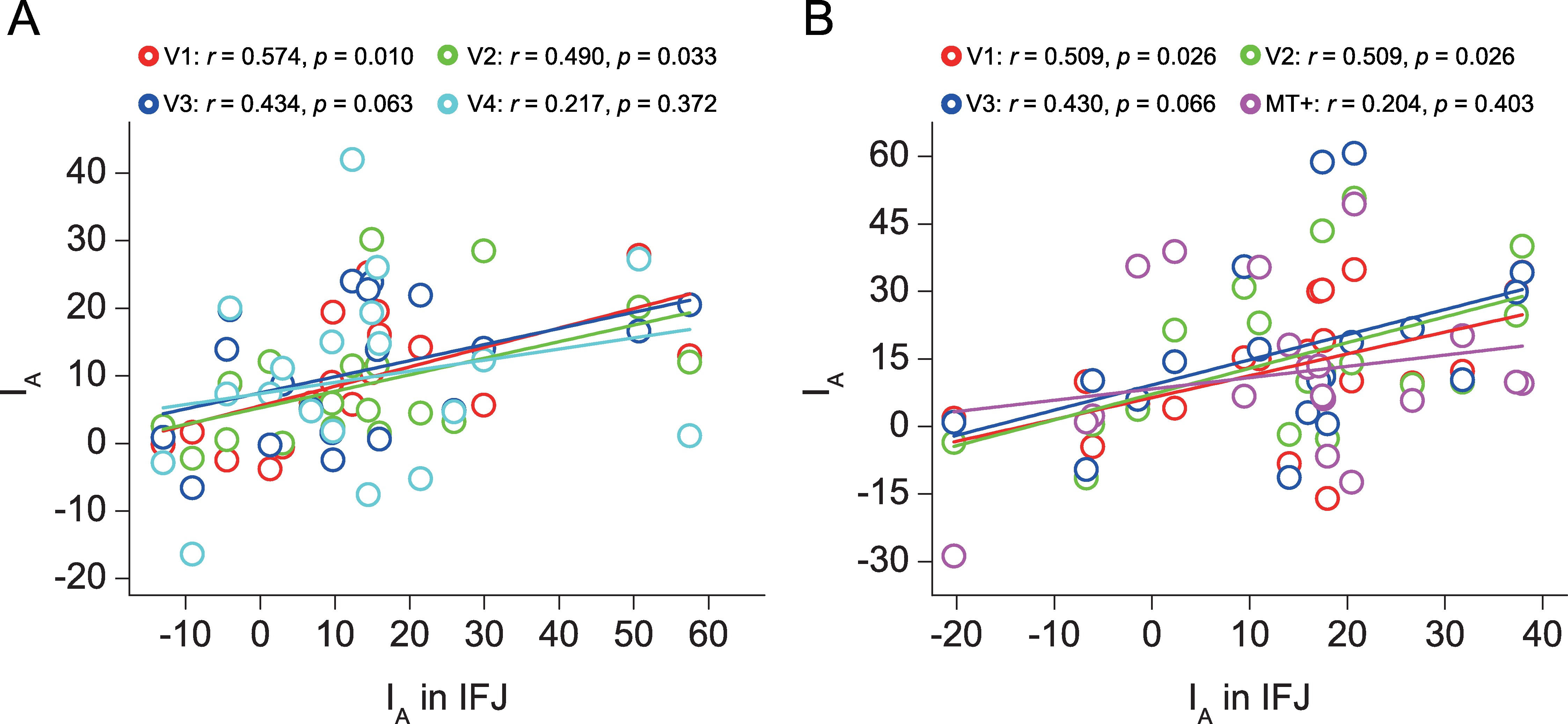

Supplement: S3 Fig — We calculated the correlation coefficients between the IA in IFJ and that in other visual processing areas across individual participants. In Experiment 1 (A), we found that the IA in IFJ correlated significantly with that in V1 (r = 0.574, p = 0.010), V2 (r = 0.490, p = 0.033), and (marginally) V3 (r = 0.434, p = 0.063), but not with that in V4 (r = 0.217, p = 0.372). In Experiment 2 (B), the IA in IFJ correlated significantly with that in V1 (r = 0.509, p = 0.026), V2 (r = 0.509, p = 0.026), and (marginally) V3 (r = 0.430, p = 0.066), but not with that in MT+ (r = 0.204, p = 0.403). These correlation analyses suggest that the spatially global effect of feature-based attention in V1–V3 may derive from feedback projections from IFJ in both experiments. Data are available from the Open Science Framework (https://osf.io/8gqk6/). IA, attentional modulation index; IFJ, inferior frontal junction; MT+, middle temporal area; V1, primary visual cortex. (TIF) [file pbio.2005399.s003.tif]

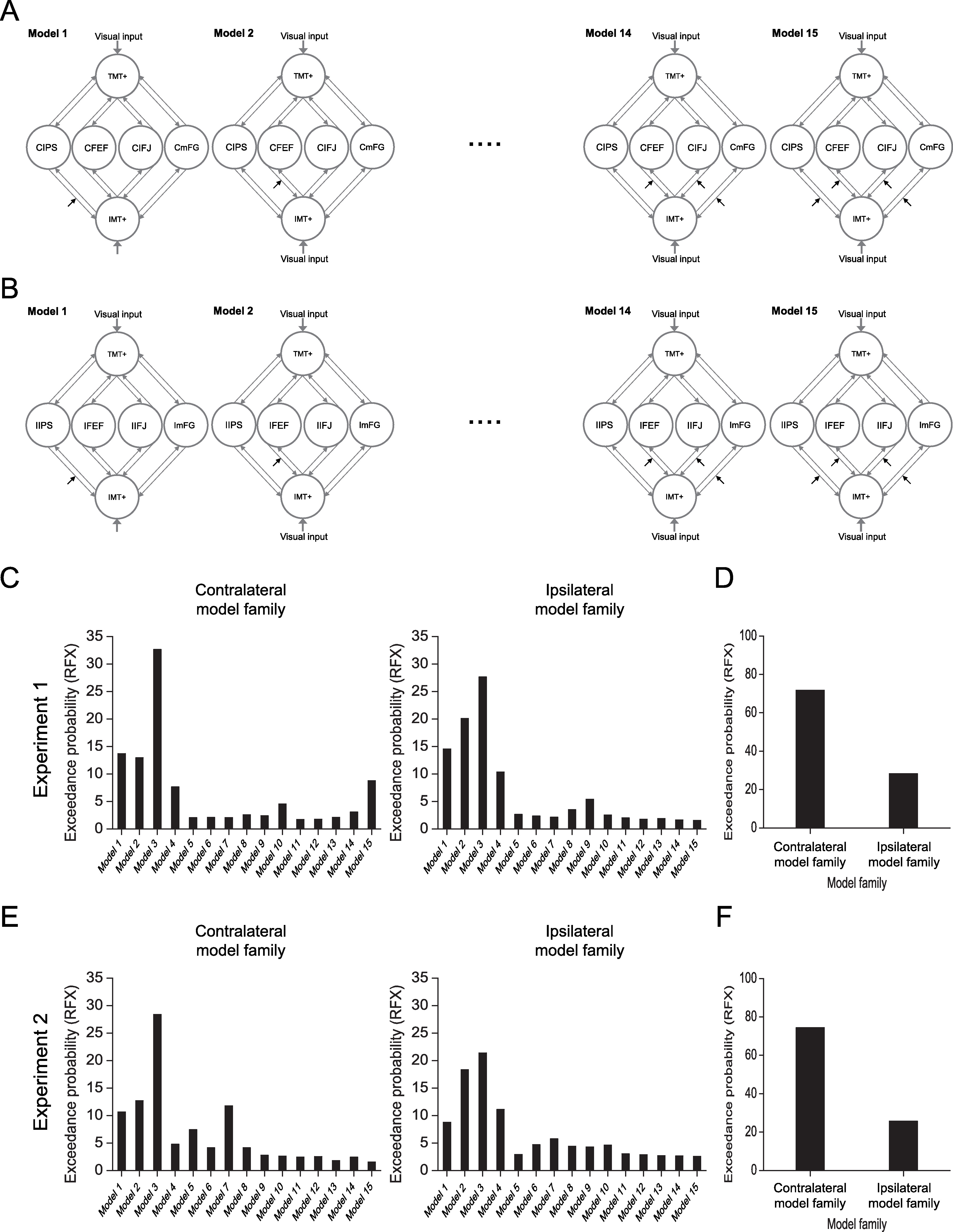

Supplement: S4 Fig — (A) The contralateral model family: each model (i.e., Models 1–15) was the corresponding model from Fig 4 with IPS, FEF, IFJ, and mFG in the hemisphere contralateral to the ignored side. CIPS, CFEF, CIFJ, and CmFG: ROI of contralateral IPS, FEF, IFJ, and mFG to the ignored side, respectively. (B) The ipsilateral model family: each model with IPS, FEF, IFJ, and mFG in the hemisphere ipsilateral to the ignored side. IIPS, IFEF, IIFJ, and ImFG: ROI of ipsilateral IPS, FEF, IFJ, and mFG to the ignored side, respectively. We applied a Bayesian model [50] comparison to select the model with the highest exceedance probability within each model family (model-level inference) and the model family with the highest exceedance probability (family-level inference). Within each model family (i.e., the contralateral and ipsilateral model families), the results showed that Model 3 was the best one to explain the modulatory effect in the Same condition in both Experiment 1 (exceedance probabilities of Models 1–15, the contralateral model family: 13.70%, 12.96%, 32.64%, 7.65%, 2.05%, 2.08%, 2.07%, 2.57%, 2.37%, 4.53%, 1.70%, 1.76%, 2.11%, 3.06%, and 8.75%, respectively; the ipsilateral model family: 14.54%, 20.11%, 27.66%, 10.36%, 2.66%, 2.34%, 2.14%, 3.49%, 5.40%, 2.53%, 2.00%, 1.74%, 1.87%, 1.63%, and 1.53%, respectively [C]) and Experiment 2 (exceedance probabilities of Models 1–15, the contralateral model family: 10.65%, 12.69%, 28.37%, 4.77%, 7.43%, 4.13%, 11.76%, 4.13%, 2.75%, 2.62%, 2.44%, 2.54%, 1.79%, 2.41%, and 1.52%, respectively; the ipsilateral model family: 8.77%, 18.33%, 21.39%, 11.11%, 2.90%, 4.68%, 5.75%, 4.41%, 4.29%, 4.62%, 3.03%, 2.86%, 2.68%, 2.63%, and 2.55%, respectively, [E]). These results further confirmed our results that the spatially global effect of feature-based attention in MT+ (Experiment 1) and V4 (Experiment 2) was derived by feedback from IFJ rather than from IPS, FEF, or mFG. Moreover, we found that the contralateral model family had a higher exce [file pbio.2005399.s004.tif]

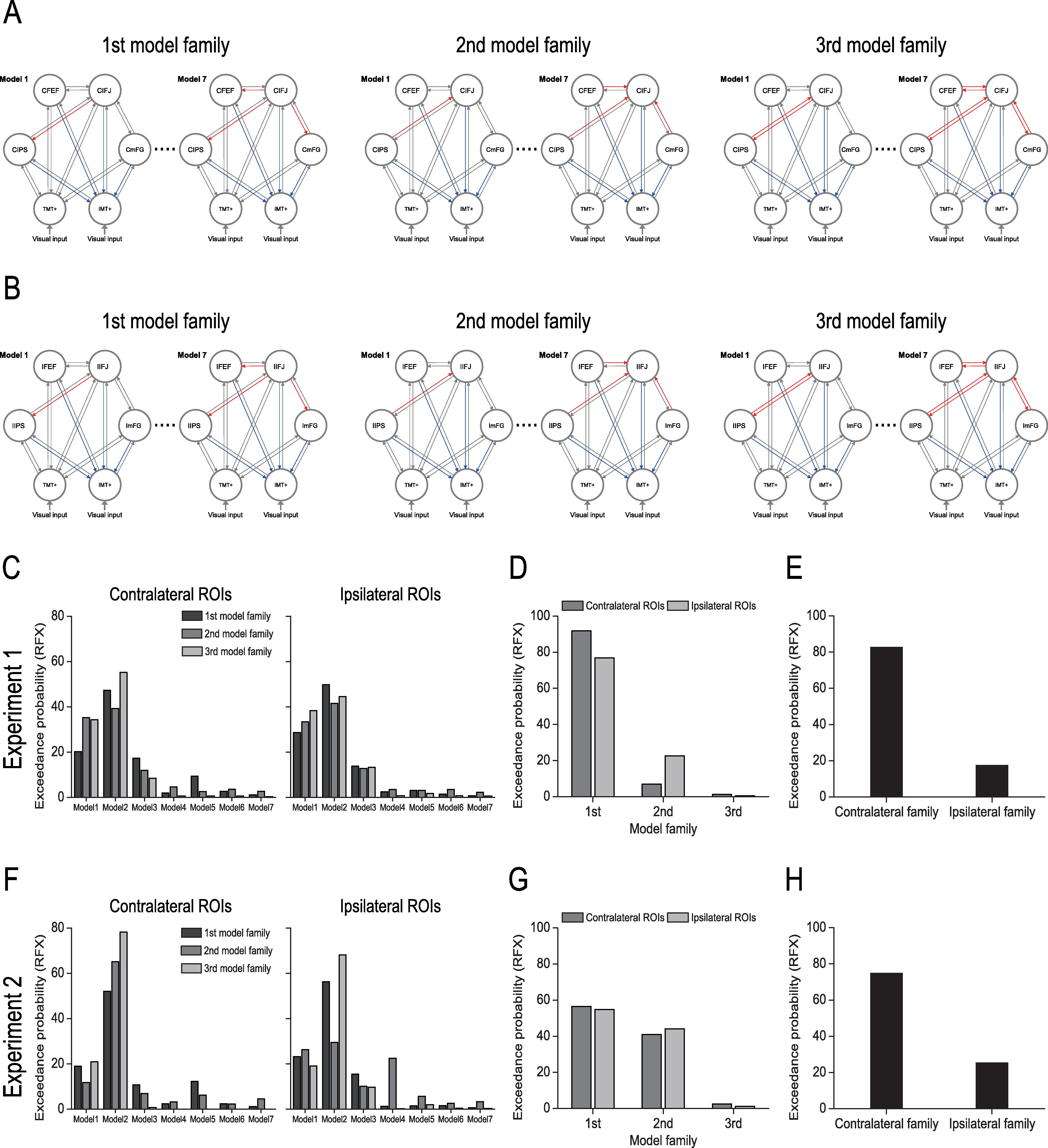

Supplement: S5 Fig — (A) The contralateral model family: each model (i.e., Models 1–7) and sub-model family (i.e., the first, second, and third model families) was the corresponding model and model family from Fig 5 with IPS, FEF, IFJ, and mFG in the hemisphere contralateral to the ignored side. CIPS, CFEF, CIFJ, and CmFG: ROI of contralateral IPS, FEF, IFJ, and mFG to the ignored side, respectively. (B) The ipsilateral model family: each model and sub-model family with IPS, FEF, IFJ, and mFG in the hemisphere ipsilateral to the ignored side. IIPS, IFEF, IIFJ, and ImFG: ROI of ipsilateral IPS, FEF, IFJ, and mFG to the ignored side, respectively. We applied a Bayesian model [50] comparison to select the model with the highest exceedance probability within each sub-model family (model-level inference) and the sub-model family with the highest exceedance probability (sub-family-level inference). We also applied a Bayesian model to compare the exceedance probability between the contralateral and ipsilateral model families. For the contralateral ROIs, within each sub-model family, the results showed that Model 2 was the best one to explain the modulatory effect in the Same condition in both Experiment 1 (exceedance probabilities of Models 1–7, the first model family: 20.23%, 47.26%, 17.34%, 1.94%, 9.39%, 2.71%, and 1.13%, respectively; the second model family: 35.24%, 39.28%, 11.89%, 4.67%, 2.61%, 3.61%, and 2.70%, respectively; the third model family: 34.32%, 55.19%, 8.40%, 0.58%, 0.61%, 0.65%, and 0.25%, respectively [C], left) and Experiment 2 (exceedance probabilities of Models 1–7, the first model family: 18.96%, 52.06%, 10.73%, 2.38%, 12.31%, 2.42%, and 1.14%, respectively; the second model family: 11.73%, 65.08%, 6.98%, 3.22%, 6.19%, 2.25%, and 4.55%, respectively; the third model family: 20.88%, 78.18%, 0.75%, 0.06%, 0.06%, 0.05%, and 0.02%, respectively [F], left). Similar results were found for the ipsilateral ROIs; within each sub-model family, Model 2 was the best one to explain [file pbio.2005399.s005.tif]

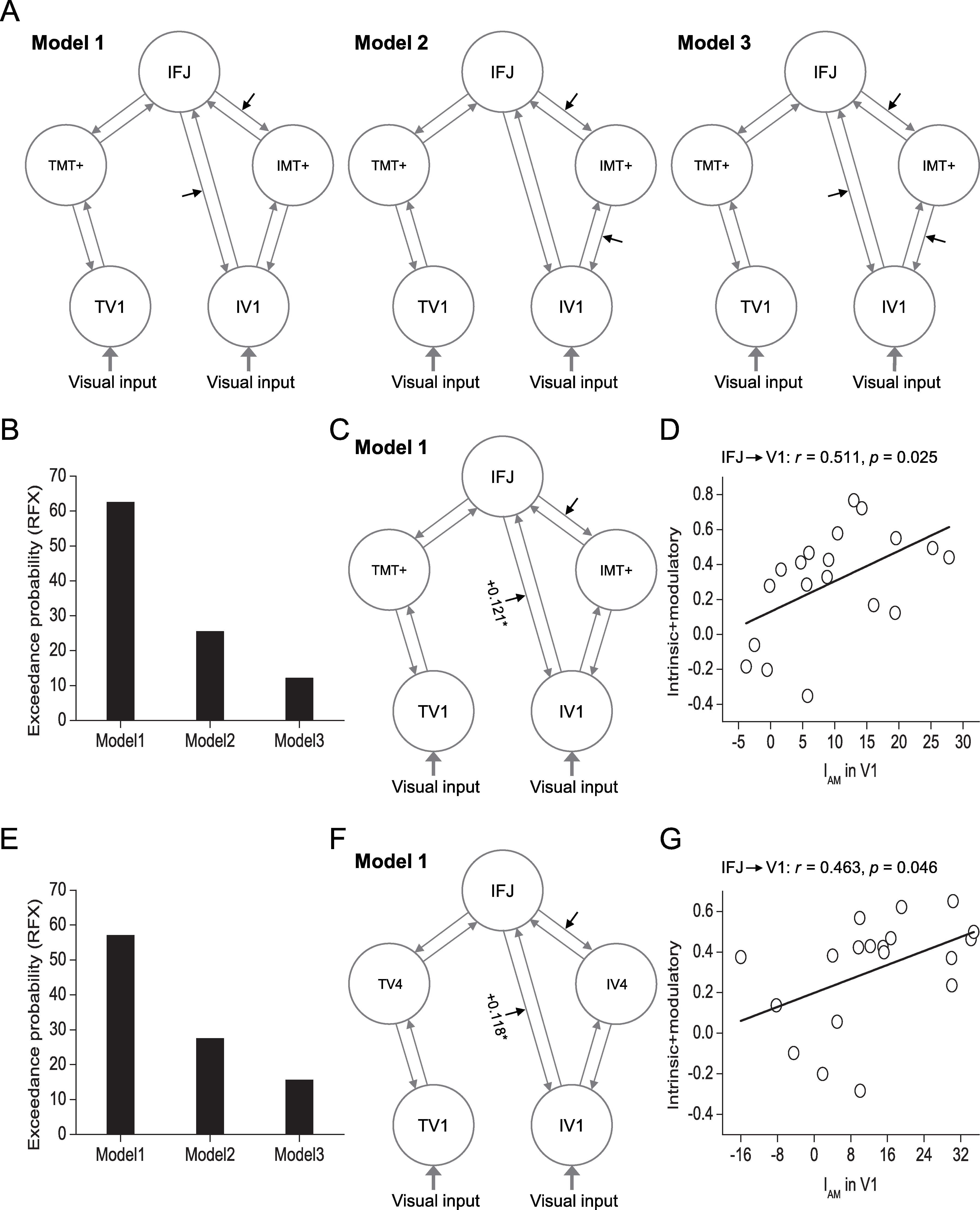

Supplement: S6 Fig — We examined whether the spatially global effect of feature-based attention in V1 is dependent on feedback from IFJ or from MT+ and V4 in Experiments 1 and 2, respectively. To examine this issue, we further defined three different models with the modulatory input (the Same condition [A]). The modulatory input could affect the feedback to V1 from IFJ (Model 1), from MT+ and V4 in Experiments 1 and 2, respectively (Model 2), or from all these areas (Model 3). TV1 and IV1: ROI in V1 evoked by the stimulus in the target and ignored sides, respectively. We examined these three models for the modulatory effect by the Same condition and fit each of the three models for each participant. Using a hierarchical Bayesian approach, we compared the three models by computing the exceedance probability of each model. In the best model, we examined the modulatory effect by the Same condition. The result showed that Model 1 was the best one to explain the modulatory effect in the Same condition in both Experiment 1 (exceedance probability, Model 1: 62.50%; Model 2: 25.43%; Model 3: 12.07% [B]) and Experiment 2 (exceedance probability, Model 1: 56.99%; Model 2: 27.43%; Model 3: 15.58% [E]). The Same condition significantly increased the feedback connectivity from IFJ to V1 in both Experiment 1 (t18 = 2.703, p = 0.015 [C]) and Experiment 2 (t18 = 2.252, p = 0.037 [F]) (*p < 0.05). Across individual participants, the feedback connectivity from IFJ to V1 correlated significantly with the IA in V1 in both Experiment 1 (r = 0.511, p = 0.025 [D]) and Experiment 2 (r = 0.463, p = 0.046 [G]). These results indicate that the spatially global effect of feature-based attention in V1 is dependent on feedback from IFJ rather than MT+ (Experiment 1) or V4 (Experiment 2). Data are available from the Open Science Framework (https://osf.io/8gqk6/). DCM, dynamic causal modeling; IA, attentional modulation index; IFJ, inferior frontal junction; MT+, middle temporal area; ROI, region of interest; V1, prim [file pbio.2005399.s006.tif]

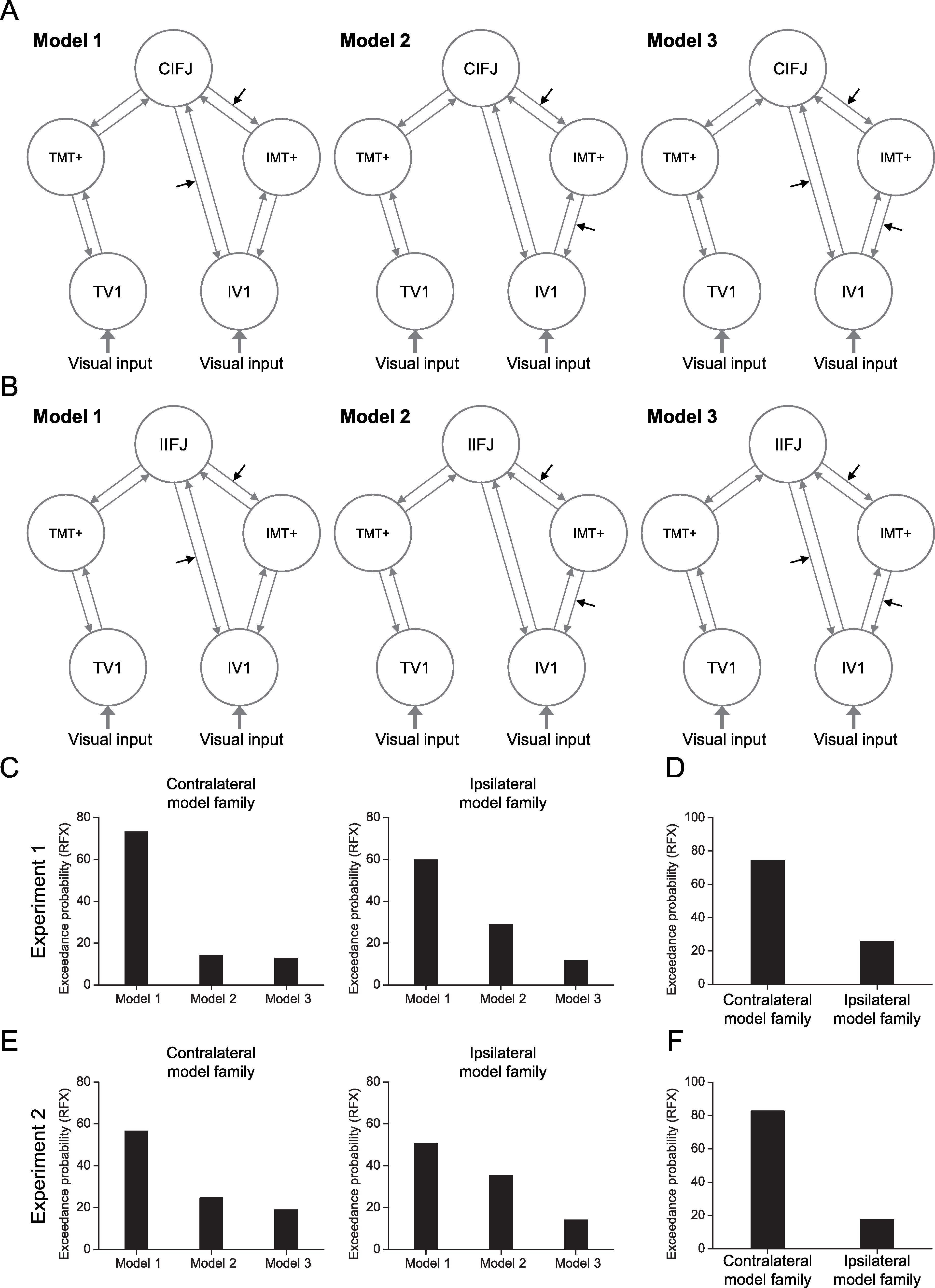

Supplement: S7 Fig — (A) The contralateral model family: each model (i.e., Models 1–3) was the corresponding model from S6 Fig with IFJ in the hemisphere contralateral to the ignored side. CIFJ: the ROI of contralateral IFJ to the ignored side. (B) The ipsilateral model family: each model with IFJ in the hemisphere ipsilateral to the ignored side. IIFJ: the ROI of ipsilateral IFJ to the ignored side. We applied a Bayesian model [50] comparison to select the model with the highest exceedance probability within each model family (model-level inference) and the model family with the highest exceedance probability (family-level inference). Within each model family (i.e., the contralateral and ipsilateral model families), the results showed that Model 1 was the best one to explain the modulatory effect in the Same condition in both Experiment 1 (exceedance probabilities of Models 1–3, the contralateral model family: 73.07%, 14.13%, and 12.80%, respectively; the ipsilateral model family: 59.72%, 28.77%, and 11.51%, respectively [C]) and Experiment 2 (exceedance probabilities of Models 1–3, the contralateral model family: 56.57%, 24.56%, and 18.87%, respectively; the ipsilateral model family: 50.61%, 35.31%, and 14.08%, respectively [E]). These results further confirmed our results that the spatially global effect of feature-based attention in V1 is dependent on feedback from IFJ rather than MT+ (Experiment 1) or V4 (Experiment 2). Moreover, we found that the contralateral model family had a higher exceedance probability than the ipsilateral model family in both Experiment 1 (exceedance probability, the contralateral model family: 74.24%; the ipsilateral model family: 25.76% [D]) and Experiment 2 (exceedance probability, the contralateral model family: 82.64%; the ipsilateral model family: 17.36% [F]). These results indicate a more crucial role of feedback from the contralateral IFJ than the ipsilateral IFJ in the spatially global effect of feature-based attention in V1 in both experiments. Da [file pbio.2005399.s007.tif]

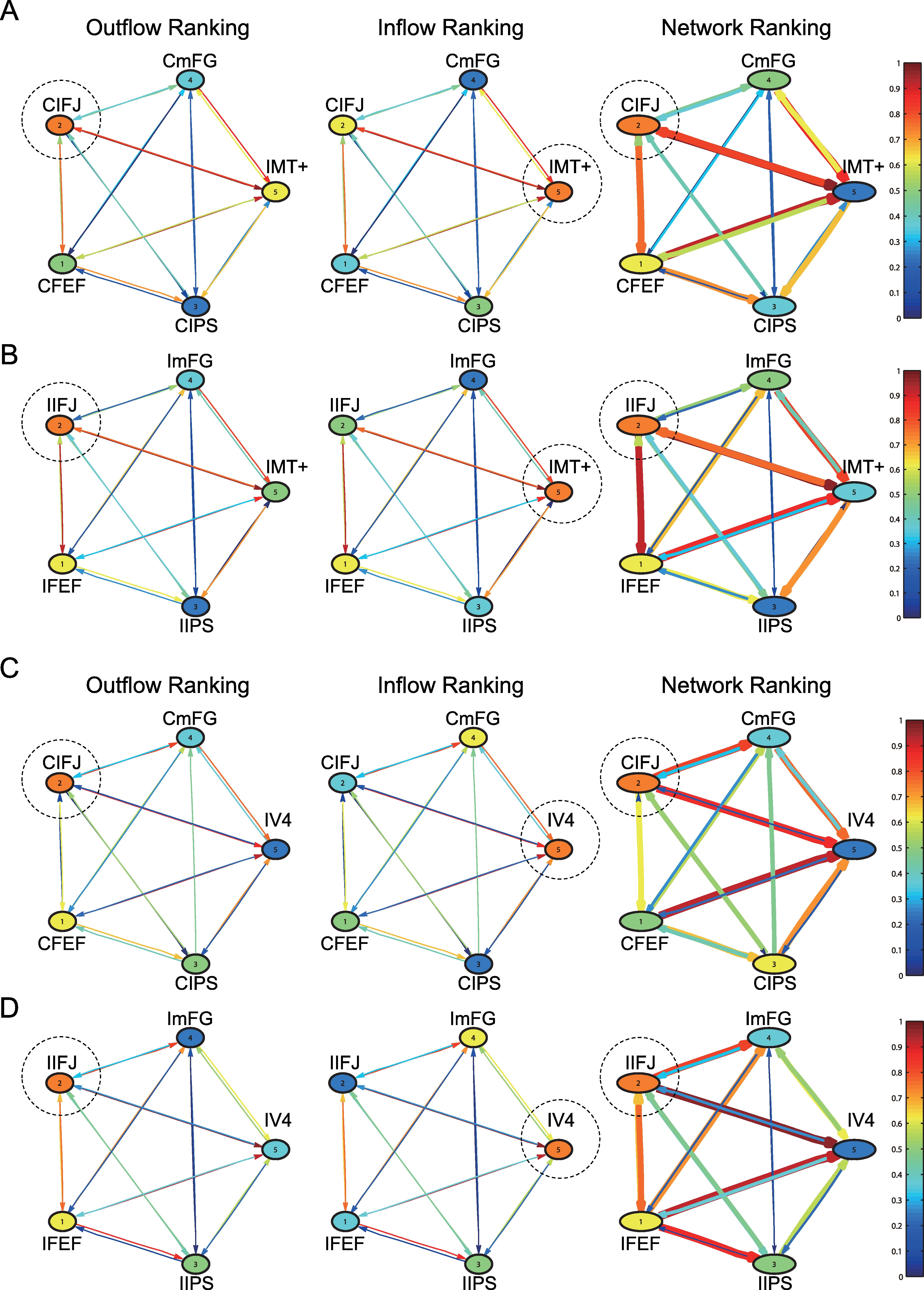

Supplement: S8 Fig — It is well known that a general concern about DCM is the problem of its hypothesis-driven approach. Thus, we used GCM [52], a data-driven approach, to further examine which area was a potential source of the spatially global effect for feature-based attention in both experiments. Unlike DCM, GCM does not require any a priori prespecification about the connectivity structure, because it studies the temporal precedence among fMRI time series using the concept of Granger causality [52], and it has been applied in numerous brain imaging studies regarding the effective connectivity analysis [82–88]. In our study, we used the GMAC toolbox (http://selene.bioing.polimi.it/BBBlab/GMAC), BIOSIG toolbox (http://biosig.sourceforge.net/), and MARSeille Boîte À Région d’Intérêt (MarsBaR) toolbox (http://marsbar.sourceforge.net/) [89] in SPM12 to our fMRI data in both experiments. First, nodes definition: all the nodes of the network were the active clusters from a group GLM analysis that showed a higher response in the Same condition than the Different condition at a significance level of p < 0.05 (corrected by FDR correction [48]) in two experiments. In Experiment 1, the MNI coordinates of the first cluster in the left and right hemispheres were [−38.4, −4.89, 38.7] and [42.1, −1.49, 39.8], respectively; those of the second were [−29.9, 25.2, 28.4] and [36.8, 25.9, 31.2], respectively; those of the third were [−28.6, −64.8, 32] and [26.9, −68.6, 36.9], respectively; those of the fourth were [−6.04, −0.326, 54.1] and [2.09, 3.81, 54.2], respectively; and those of the fifth were [−40, −70, −6.11] and [39, −68.9, −8.69], respectively. The first to fifth clusters were localized in bilateral FEF, IFJ, IPS, mFG, and MT+, respectively, and they were defined as Nodes 1 through 5, respectively (A and B). In Experiment 2, the MNI coordinates of the first cluster in the left and right hemispheres were [−40.6, −3.50, 38.7] and [42.2, 0.317, 41.2], respectively; those of the second were [−31 [file pbio.2005399.s008.tif]
